# Additive Manufactured Magnetoelectric Wireless Retainer as the Periodontal Remodeler for Post‐Orthodontic Tissue Reconstruction and Relapse‐Inhibition

**DOI:** 10.1002/advs.202505020

**Published:** 2025-07-23

**Authors:** Haoqi Lei, Jiwei Sun, Peng Chen, Li Hu, Xin Zhou, Shiqiang Gong, Qingming Tang, Xiaoqing Han, Lili Chen, Bin Su

**Affiliations:** ^1^ Department of Stomatology Union Hospital, Tongji Medical College Huazhong University of Science and Technology Wuhan 430022 China; ^2^ School of Stomatology Tongji Medical College Huazhong University of Science and Technology Wuhan 430030 China; ^3^ Hubei Province Key Laboratory of Oral and Maxillofacial Development and Regeneration Wuhan 430022 China; ^4^ State Key Laboratory of Materials Processing and Die & Mould Technology School of Materials Science and Engineering Huazhong University of Science and Technology Wuhan 430074 China; ^5^ Department of Stomatology Tongji Hospital Tongji Medical College Huazhong University of Science and Technology Wuhan 430030 China

**Keywords:** additive manufacturing, periodontal remodel, post‐orthodontic retention, wireless magnetoelectric

## Abstract

Modern orthodontics since 1899 has failed to solve rapid and persistent dentition maintenance after tooth movement till now. Traditional post‐orthodontic retention using single mechanical fixation cannot actuate periodontal tissue remodel, causing lifelong dependence, huge time costs and discomforts. Current attempts by combining hormone drug with mechanical fixation for improvement are limited by unsatisfactory outcomes and global side effects. Targeting at this 120‐year dilemma, a flexible magnetoelectric orthodontic retainer (FMOR) is presented using customized additive manufacturing, serving as periodontal remodeler for high‐performance post‐orthodontic retention. Wireless magnetoelectric cues display considerable effects in periodontal microenvironment remodel, switching periodontal ligament cell (PDLC) paracrine manner to promote M2 macrophage formation, osteoclastic/osteoblastic balance and collagen metabolism. Using rat and rabbit orthodontic model, application of FMOR is confirmed to significantly strengthen the maintaining of tooth movement outcomes. Mechanistically, magnetoelectric treatment significantly upregulate PDLC genes related to immune response, extracellular matrix organization and osteogenic regulation. This study proposes a non‐destructive physical therapeutic strategy for fast activation of periodontal reconstruction after orthodontic treatment and inhibition of tooth relapse for the first time, shedding light on development of next‐generation orthodontic retention system for optimized solution of orthodontic relapse.

## Graphical abstract

Orthodontic relapse persists as a 120‐year challenge, as traditional retainers fail to remodel tissue, causing lifelong dependence. This study demonstrates a flexible magnetoelectric retainer (FMOR) enabling wireless periodontal remodeling. FMOR cues shift periodontal ligament cell (PDLC) paracrine signaling, promoting M2 macrophages, osteogenic balance, and collagen metabolism. Rat/rabbit models confirm FMOR significantly strengthens tooth position maintenance, proposing a non‐destructive physical strategy against relapse and enabling next‐generation retention systems.

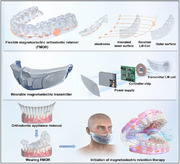

## 1 Introduction

Malocclusion serves as a common abnormality of dentition and maxillofacial bone that causes great discomfort and secondary disease risks to patients.^[^ ^1^ ^]^ Modern orthodontic treatment has been developed to rectify malocclusion using biomechanical induction over a century.^[^ ^2^ ^]^ Nevertheless, how to maintain the achieved orthodontic effect still lies as an unsolved difficulty.^[^ ^3^, ^4^ ^]^ It should be noted that teeth are guided to a desired position after finishing an orthodontic course. In this case, periodontal tissues have not been transformed from aseptic inflammatory status to pro‐reconstructing manner, which is mainly explained by restored stress within the periodontal ligament (PDL) against opposite orthodontic force.^[^ ^5^, ^6^ ^]^ To rebuild the periodontal tissues around the orthodontic teeth, traditional mechanical retainer is advocated to patients for permanent passive retention.^[^ ^7^, ^8^ ^]^ However, this is a huge burden to post‐treatment patients owing to its significant demand of time and discomfort.^[^ ^9^ ^]^ Once abandoning to persist in mechanical retention, the periodontal tissues fail to recover, leading to rapid reverse of orthodontic‐modified dentition due to instable periodontal status.^[^ ^10^, ^11^ ^]^ Therefore, key for this dilemma lies in the disability of active regulation on periodontal microenvironment. Post‐orthodontic periodontal tissues fail to get guided to perform repair and reconstruction, making it especially susceptible to relapse in response to external stimulus.^[^ ^12^, ^13^, ^14^ ^]^ In summary, rapid periodontal microenvironment remodel for orthodontic dentition is of great importance.

To solve this problem, orthodontists have explored collaboration of mechanical fixation and drug delivery to enhance post‐orthodontic retention.^[^ ^15^, ^16^, ^17^ ^]^ Supplementation of hormone drugs, such as parathyroid hormone and atorvastatin, has been identified to promote the stability of post‐orthodontic dentition by enhancing periodontal remodel, including the counts of osteoclasts and osteoblasts, as well as expression of bone metabolic proteins.^[^ ^15^, ^16^ ^]^ However, global administration of drugs poses some unnecessary side effects, and deficiency in targeting to periodontal tissues would lead to inadequate accumulation of effective constituents in the local area, further compromising its clinical expectations. Local application thus acts a proper alternative, but surface dressing is difficult to penetrate into periodontal ligaments.^[^ ^17^ ^]^ Repetitive injections would bring about sufferings and inconvenience to patients, and this invasive operation is also harmful to periodontal remodel.^[^ ^18^, ^19^ ^]^ In consequence, current studies still failed to provide an optimal strategy for active and stable remodel of periodontal tissues and tooth positions.

Biophysical electric cues have been identified to play an indispensable role in tissue development, homeostasis and regeneration.^[^ ^20^, ^21^, ^22^, ^23^ ^]^ Endogenous bioelectric fields regulate key regenerative processes including osteogenesis, angiogenesis, and immunomodulation through voltage‐gated ion channels and electrophysiological signaling pathways.^[^ ^24^, ^25^ ^]^ Although mild local irritation and thermal effects may occur at high intensities, clinical applications of exogenous bioelectric stimulation demonstrate accelerated tissue repair with minimal side effects compared to pharmacological interventions, owing to its non‐thermal, non‐invasive nature.^[^ ^26^, ^27^ ^]^ In particular, we previously confirmed that exogenous supplementation of electric signals would benefit repair of nerve, skin and especially maxillofacial bone tissues.^[^ ^28^, ^29^, ^30^ ^]^ Therefore, it is speculated that bio‐electric signals might promote remodel of periodontal tissues. Meanwhile, electric stimulation could be a non‐destructive method with high‐penetration for biological intervention, which is suitable for daily intervention post orthodontic treatment.^[^ ^31^, ^32^ ^]^ Nevertheless, in orthodontic retention situation, the whole setup (electrodes, wires and power sources) is rigid, unable to form superior adaptation with soft tissues.^[^ ^33^, ^34^ ^]^ Limited by definite intraoral space, integration of inner battery, wires and electrodes into mechanical retainers remains largely impractical, and might cause potential inconvenience and risks.^[^ ^35^, ^36^ ^]^ Hence, development of novel wearable orthodontic retainers with built‐in electrodes activated by external power exhibits huge value. Flexible powering method based on Faraday's law of electromagnetic transformation might be a potential solution for relapse inhibition, whose advantages include wireless energy output, adequate current generation and precise control in intensity.^[^ ^37^, ^38^ ^]^ Besides, taking advantage of the combination of additive manufacturing and dental CT scanning, customized flexible magnetoelectric device can be designed and fabricated on basis of personal difference in anatomic morphology.^[^ ^39^, ^40^ ^]^

**Figure 1:**
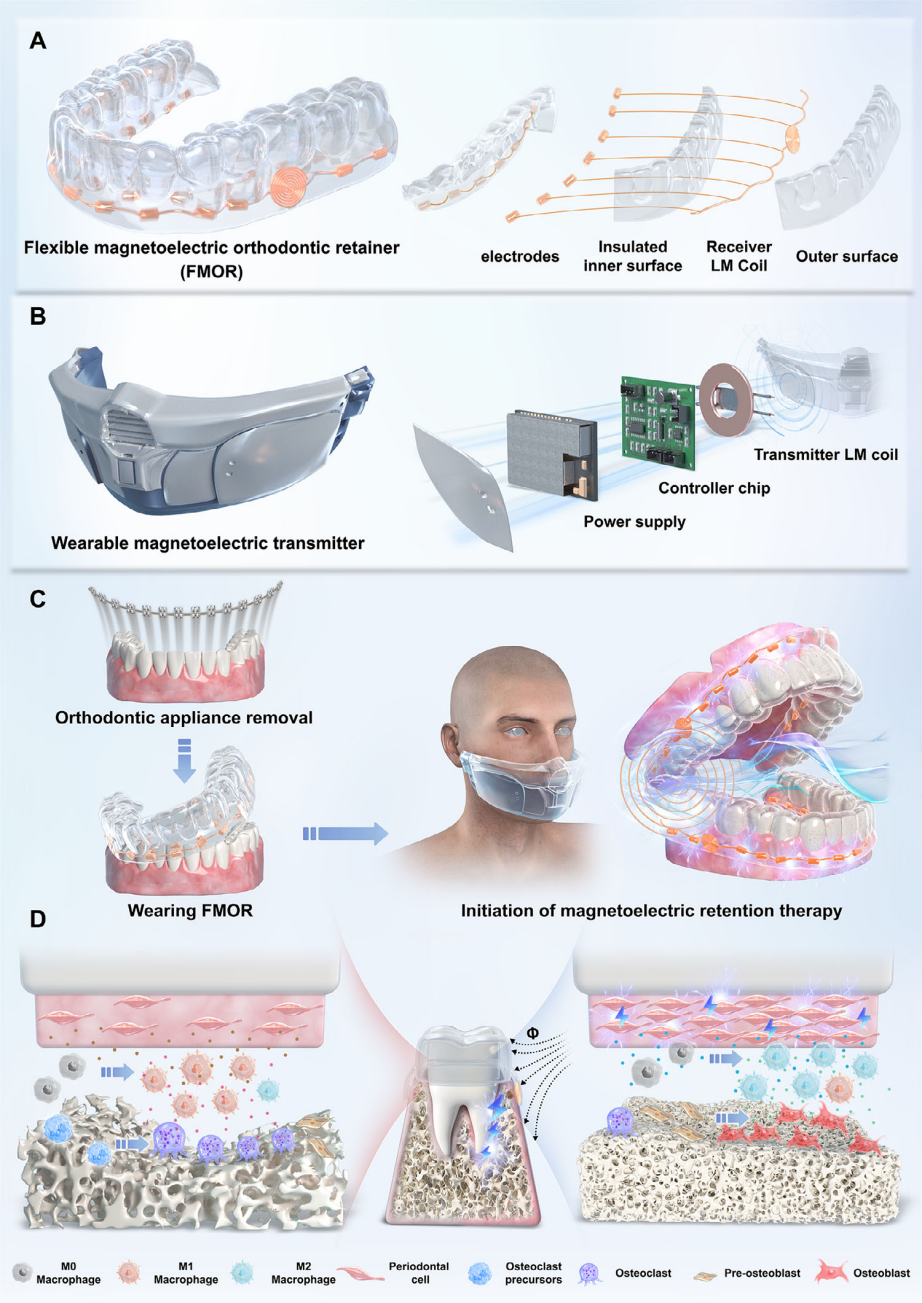
Schematic illustration of the FMOR system and the post‐orthodontic tissue reconstruction. A) The flexible magnetoelectric orthodontic retainer and its components. B) The wearable magnetoelectric transmitter and its disassembled electronic structure. C) The activation of magnetoelectric retention therapy at the end of orthodontic treatment. D) The magnetoelectric cues promote immune repair and tissue reconstruction in periodontal tissues to inhibit orthodontic relapse.

Here, we demonstrated a novel flexible magnetoelectric orthodontic retainer (FMOR) that could maintain more than 60% tooth movement outcomes, which reached almost twofold compared to pure mechanical retention group, while the dentition lost almost the whole orthodontic effect in the without retention 10 days after treatment. The FMOR consists of a soft retainer backbone, liquid metal (LM) coils that enables electric output in response to external alternating magnetic field and bio‐interfaces for electric transmission into periodontal tissues (**Figure** **1**A–C). We identified that magnetoelectric output from FMOR can remodel the orthodontic force‐induced pro‐inflammatory periodontal microenvironment. In detail, magnetoelectric cues dominate the transformation of periodontal ligament cell (PDLC) paracrine manner from stress‐induced status, thus triggering M2 macrophage polarization, re‐balancing osteoclastic and osteoblastic activity and facilitating collagen deposition (Figure 1D). Mechanistic exploration delineates that magnetoelectric retention treatment significantly upregulated the genes related to immune response, extracellular matrix organization, and osteogenesis regulation in PDLC. Application of wireless magnetoelectric intervention achieved significant effect in inhibition of post‐orthodontic relapses and reconstruction of periodontal tissues in rat and rabbit models, evidenced by augmented alveolar bone formation and collagen deposition.

## 2 Results and Discussion

### 2.1 Design and Fabrication of FMOR System

**Figure 2:**
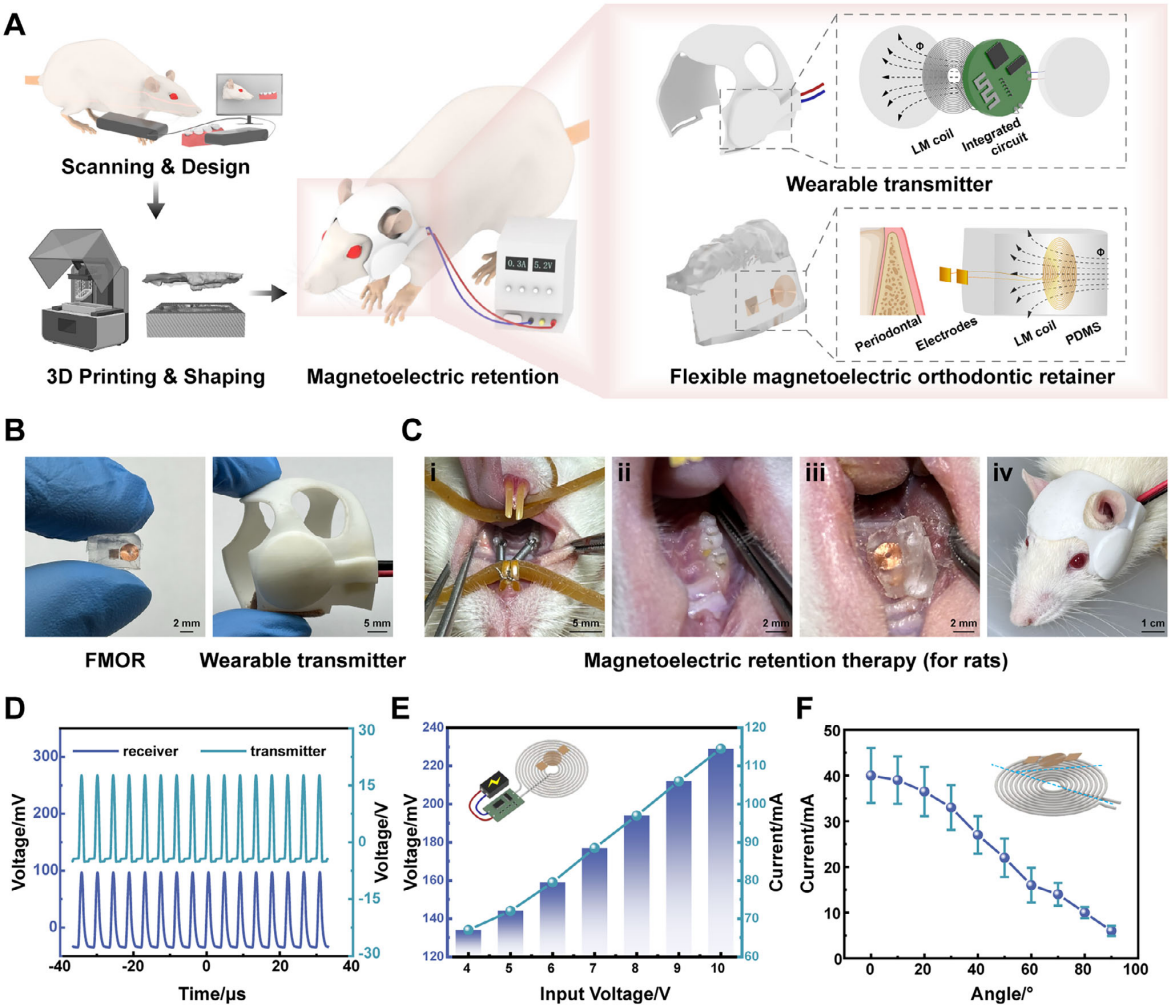
Fabrication and characterization of the FMOR system. A) Schematic diagram of the fabrication process and the components of FMOR and the wearable magnetoelectric transmitter. B) An optical image of FMOR and the wearable magnetoelectric transmitter for rat experiments. C) Magnetoelectric retention therapy for rats in vivo: i) Moving the rat first molar to the target position after the OTM phase. ii) Wearing personalized FMORs. iii) Initiating magnetoelectric retention therapy with the wearable magnetoelectric transmitter. D) Voltage waveforms at FMOR receiver and wearable magnetoelectric transmitter. E) Electrical output of different input voltages between the receiver and transmitter. F) Current output at different angels (θ) between FMOR receiving coil and the transmitting coil.

The receiving and transmitting units of the FMOR system were designed and constructed as shown in **Figure** **2**A. Briefly, a 3D scanner was used to capture the individual teeth and craniomaxillofacial recordings into a digital model. The personalized FMOR and wearable transmitter were designed by simulation design software, and the molded parts were finally printed and encapsulated with LM coils by a light‐curing 3D printing equipment. The retainers molded from polydimethylsiloxane (PDMS) are biocompatible, ensuring that the FMOR, as the receiving end, is harmless during application.^[^ ^41^ ^]^ The rationale behind the choice of this flexible material is due to its elasticity and toughness, which permit the retainer to have a certain degree of physical retention based on improving the comfort of the patient's wear, as well as its capacity to stably and safely encapsulate the flexible LM coils and circuit structure inside (Figure 2B). The overall dimensions of the FMOR are ≈6.2 × 5.6 × 4.3 mm^3^. And FMOR adopts a unified monolithic design with three integrated elements (Figure S1A, Supporting Information):

1. PDMS substrate: Encapsulates components while serving as fixation;
2. Embedded copper coil: Positioned within PDMS at gingival interface;
3. Surface‐mounted electrode: Adhered to PDMS and wire‐connected to coil.

This optimized design minimizes the intraoral footprint, which directly addresses the spatial constraints mentioned in the introduction, while ensuring mechanical robustness through complete component encapsulation. Concurrently, in order to guarantee the precise delivery of wireless magnetoelectric cues to the periodontal, the electrodes were positioned on the inner side of the FMOR and securely attached to the gums, while the remainder of the internal wiring was insulated from the oral cavity (Figure S1B, Supporting Information). Enhanced cross‐sectional schematics and images of FMOR worn on the rat maxillary specimens captured with a magnifying lens head demonstrated the contact interface between the FMOR electrodes and the periodontal tissue (Figure S1C, Supporting Information, Figure 2C). Results of Young's modulus showed that FMOR integrated with LM coil can meet the strength requirements for intra‐oral wearing (Figure S4, Supporting Information). Then, we further compared the measured Young's modulus with that of currently used medical‐grade materials (Table S2, Supporting Information). The results indicate that the PDMS used in this work falls within the typical range for flexible transparent materials. The most commonly used material for medical retainers is PETG,^[^ ^42^ ^]^ which has a Young's modulus of up to 2.2 GPa. In comparison, our PDMS exhibits significantly better flexibility, making it more suitable for wearable and biomedical applications.

The wearable transmitter was designed by craniomaxillofacial 3D scanning data. The core component of the wearable magnetoelectric transmitter was the integrated circuit and the LM transmitter coil. The integrated circuit can monitor and regulate current output parameters of the system in real time (Figure 2B, Figure S2, Supporting Information). Similarly, the aforementioned process was utilized to develop the personalized FMOR system that was subsequently applied to rabbits (Figure S3, Supporting Information). In summary, the personalized FMOR was paired with the wearable magnetoelectric transmitter for subsequent magnetoelectric retention therapy (Figure 2C).

### 2.2 Wireless Magnetoelectric Output Performance of FMOR

FMOR is based on Faraday's law of electromagnetic transformation. The alternating current created an alternating magnetic field around the LM coil at the transmitter end, and the magnetic induction at any point in space can be approximated by the Biot‐Saval law:^[^ ^43^ ^]^

(1) $$d\vec{B}=\frac{\mu_{0}}{4\pi }\frac{Id\vec{l}\times \hat{r}}{r^{2}}$$

As the receiving end, the receiving coil in the FMOR that was under the influence of an alternating magnetic field generates an induced electromotive force within it (Figure 2D):

(2) $$\varepsilon =-\frac{\oint_{A}d\vec{B}\times d\vec{A}}{dt}$$

Subsequently, considering the spatial position of the coil is not fixed in practical use, we conducted mutual inductance experiments to regulate and evaluate the wireless magnetoelectric output performance of the FMOR. We considered that the relative position and transmitted power between the coils can significantly influence the magnetoelectric efficiency.^[^ ^44^, ^45^, ^46^ ^]^ To more accurately assess potential operational issues of the FMOR, we conducted performance tests on the mutual inductance system. By adjusting the voltage at the transmitter side, the voltage across the FMOR changes synchronously (Figure 2E, Video S1, Supporting Information), which is crucial for maintaining a stable effective output current. When the FMOR current deviates from the desired therapeutic range, it can be effectively regulated by tuning the transmitter voltage. During actual fabrication and wearing processes, it is difficult to ensure consistent alignment between the FMOR coil and the transmitting coil. Within the coverage area of the transmitting coil, variations in horizontal offset, radial distance, and angular misalignment may occur. Therefore, a series of experiments were carried out with the transmitting voltage fixed at ≈4 V. The results show that the FMOR receiver coil maintains a physiologically effective output current of ≈20 mA at distances up to 5 mm (Figure S5, Supporting Information). Moreover, an effective output current is still achieved with angular deviations of up to 40° (Figure 2F). The series of experiments demonstrated that while alterations in relative position resulted in notable alterations in the magnetoelectric output, the constructed parameters exhibited the physiological requirements. A comparison with existing studies indicates that the magnetic field strength and current applied in our experiments are within the recognized safety limits.^[^ ^47^, ^48^, ^49^ ^]^ Concurrently, real‐time temperature monitoring demonstrated that the temperature fluctuations of the transmitting and receiving LM coils during magnetoelectric retention therapy remained within the range of 37 °C, re‐ensuring the safety of FMOR system during its wireless magnetoelectric output state (Figure S6, Supporting Information).

In conclusion, preliminary in vitro experiments have demonstrated that FMOR can achieve stable physiological‐grade wireless magnetoelectric output and safe delivery to periodontal tissues through the magnetoelectric induction of a wearable magnetoelectric transmitter.

### 2.3 FMOR Inhibits Orthodontic Tooth Relapse In Vivo

**Figure 3:**
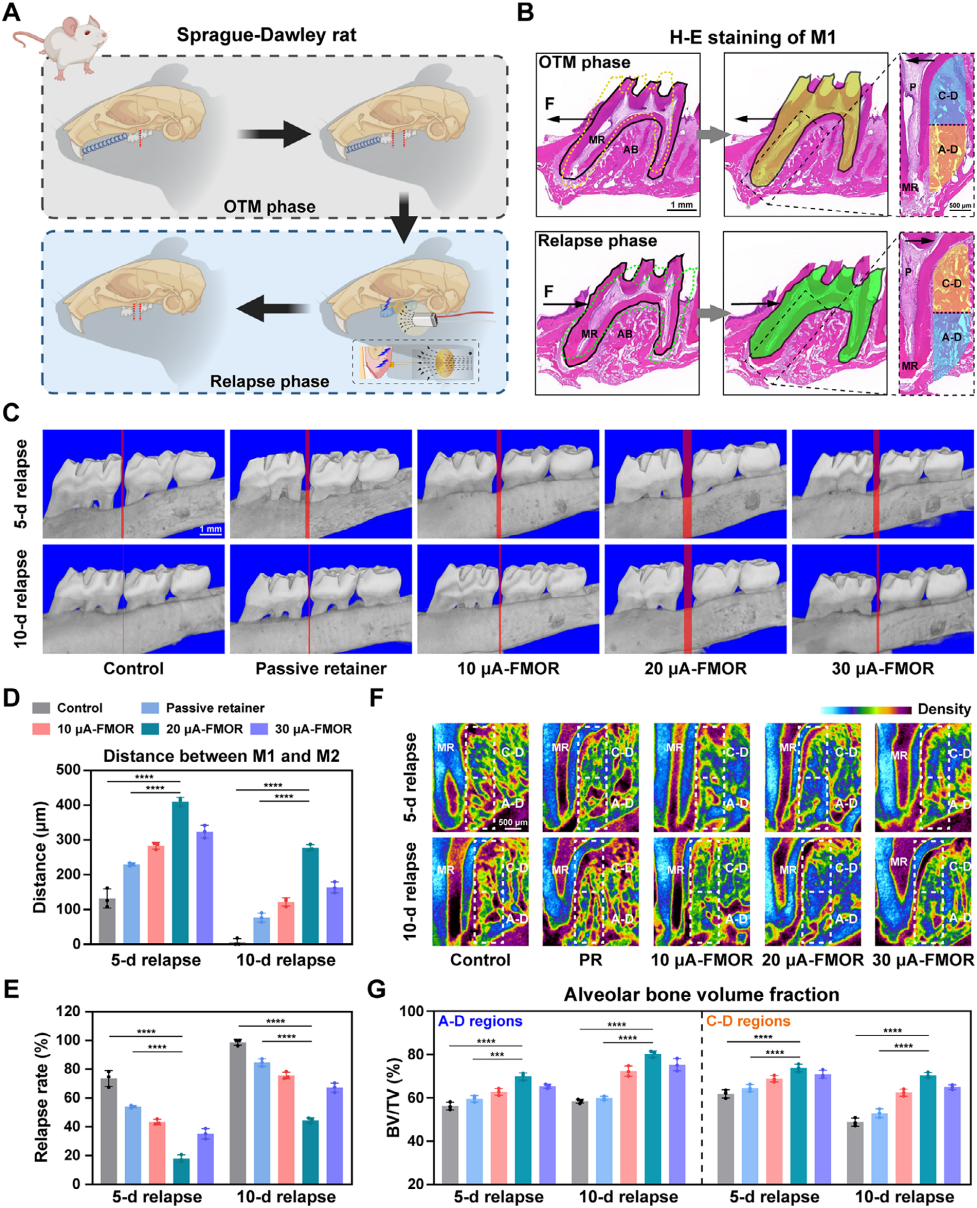
FMOR inhibits orthodontic tooth relapse in vivo. A) Schematic illustration of the orthodontic and relapse rat model. Created by BioRender.com. B) Schematic diagram of H&E staining showing regional division of the first molar during orthodontic and relapse processes. Labels: M1 the first molar, MR mesial root, AB alveolar bone, A‐D the apical distal region, C‐D the cervical distal region, P tooth pulp. The blue area indicates tension sides, the orange area indicates pressure sides. C) Representative micro‐CT images of rats’ dentition after 5 and 10 days of orthodontic relapse. The width of the red area is the distance between the first and second molars. Scale bar = 1 mm. D,E) Quantitative analysis of the distance between first and second molars and the relapse rate. Relapse rate = relapse distance / relapse distance at 0 d. F,G) Micro–computed tomography images and quantitative analysis of percent bone volume (BV/TV) of alveolar bone in the A‐D and C‐D regions. *p < 0.05, **p < 0.01, ***p < 0.001 and ****p < 0.0001. Sample size (n = 3).

The schema in **Figure** **3**A shows the experimental design in the rat relapse model, including orthodontic force application and relapse phase.^[^ ^50^ ^]^ The maxillary first molar (M1) of the rat was rapidly moved by the 40 g of optimal orthodontic force provided by a tension spring, resulting in the creation of a gap between it and the second molar (M2). This stage is referred to as the Orthodontic Movement (OTM) phase.^[^ ^51^ ^]^ Once the orthodontic force was removed, the first molar entered the relapse phase under the compressing and stretching forces of the periodontal ligament. Previous studies have verified that the type of OTM in rats is tipping due to the force was loaded on the neck of the tooth.^[^ ^51^, ^52^ ^]^ By analyzing the stress distribution of the mesial root (MR) in Figure 3B, it was shown that the periodontal tissues in the apical distal region (A‐D region) were the major area subjected to orthodontic pressure during the OTM phase, resulting in significant bone resorption. While the cervical distal region (C‐D region) was the tension side during the OTM phase, leading to bone deposition. In contrast, orthodontic relapse resulted in a completely opposite movement trajectory compared to the OTM phase. Additionally, it produced completely opposite stress distributions and tissue behaviors. In the relapse phase, C‐D region was under bone resorption in response to the compressive stress, whereas A‐D region is the tension area with bone deposition.

To explore the effect of magnetoelectric retention therapy on orthodontic relapse, during the relapse phase, rats were fitted with FMOR for 12 h daily and the wearable magnetoelectric transmitter device were adjusted to set varying magnetoelectric output gradients. Rats in the control group did not wear retainers during the relapse phase, while the passive retainer (PR) indicated that FMOR was equipped without initiating the magnetoelectric retention therapy. The FMOR group was classified into three subgroups based on current amplitude (10, 20, and 30 µA). After 5 and 10 days of relapse, Micro‐CT 3D remodeling and sagittal images of rat maxillary molars was performed and the distance between M1 and M2 was measured. The results showed that FMOR with magnetoelectric output significantly reduced the M1 relapse distance, with the most pronounced effect in the 20 µA current group (Figure 3C,D and Figure S7A, Supporting Information). The M1 relapse rate was calculated by dividing the relapse distance by the OTM distance. Results showed that the 20 µA‐FMOR therapy during the 10‐day relapse phase significantly reduced the relapse rate from 100% in control group to about 50% (Figure 3E).

In order to analyze the dynamic variation of alveolar bone density in the A‐D and C‐D regions of the MR distal region described above, we visualized the tomographic images of the alveolar bone using Micro‐CT data, which represented the level of bone density in terms of color. Results of the tomographic images and bone volume fraction (BV/TV) showed that the alveolar bone treated with magnetoelectric retention therapy achieved higher bone density, especially in the 20 µA group (Figure 3F,G). A comparison of the control and PR groups through stress distribution analysis revealed that, as the deposition side, A‐D area exhibited a more rapid osteogenesis rate during the relapse phase under 20 µA‐FMOR therapy. Conversely, C‐D area demonstrated a notable bone resorption inhibition effect as the bone resorption side under 20 µA‐FMOR therapy.

With the strong support in the distal region of the MR, and considering that the biological changes occurred throughout the alveolar bone, we further analyzed the regions of the distal roots as well. In previous studies,^[^ ^51^ ^]^ the pressure in the distal root were mainly distributed in the cervical mesial region (C‐M region, Figure S7B, Supporting Information). Based on this, we now performed a comprehensive analysis of the C‐M region of the distal root. Micro‐CT reconstruction and quantification of bone volume fraction (BV/TV) showed that the alveolar bone density was significantly higher after magnetoelectric retention treatment, especially in the 20 µA subgroup (Figure S7C, Supporting Information). The results of the distal roots further reinforced the therapeutic effect of magnetoelectric retention therapy.

Finally, it is imperative to undertake a critical evaluation to ascertain the potential contribution of the magnetic field alone. We established a dedicated magnetic field control group (MF Group) where rats wore passive retainers (PR) without liquid metal coils and was received identical 12‐h daily magnetic field exposure (20 µA‐equivalent intensity). After 5 and 10 days of relapse, Micro‐CT measurements showed no statistically significant differences in relapse distance or relapse rate between MF and PR groups (Figure S8A–D, Supporting Information). Sagittal reconstructions and BV/TV quantification in three critical regions (A‐D, C‐D, C‐M regions) revealed comparable alveolar bone density in MF and PR groups, and both groups are significantly lower than FMOR groups (Figure S8E,F, Supporting Information). These comprehensive analyses confirm that the actuating magnetic field alone exerts no measurable effect on periodontal remodeling or relapse inhibition, validating that the observed therapeutic effect of FMOR requires magnetoelectric stimulation.

Hence, FMOR effectively inhibited orthodontic tooth relapse by 20 µA‐FMOR therapy, which may be related to magnetoelectric cues modulating periodontal microenvironment during the relapse phase.

### 2.4 Magnetoelectric Output Dominate the Remodel of Periodontal Microenvironment During the Relapse Phase

**Figure 4:**
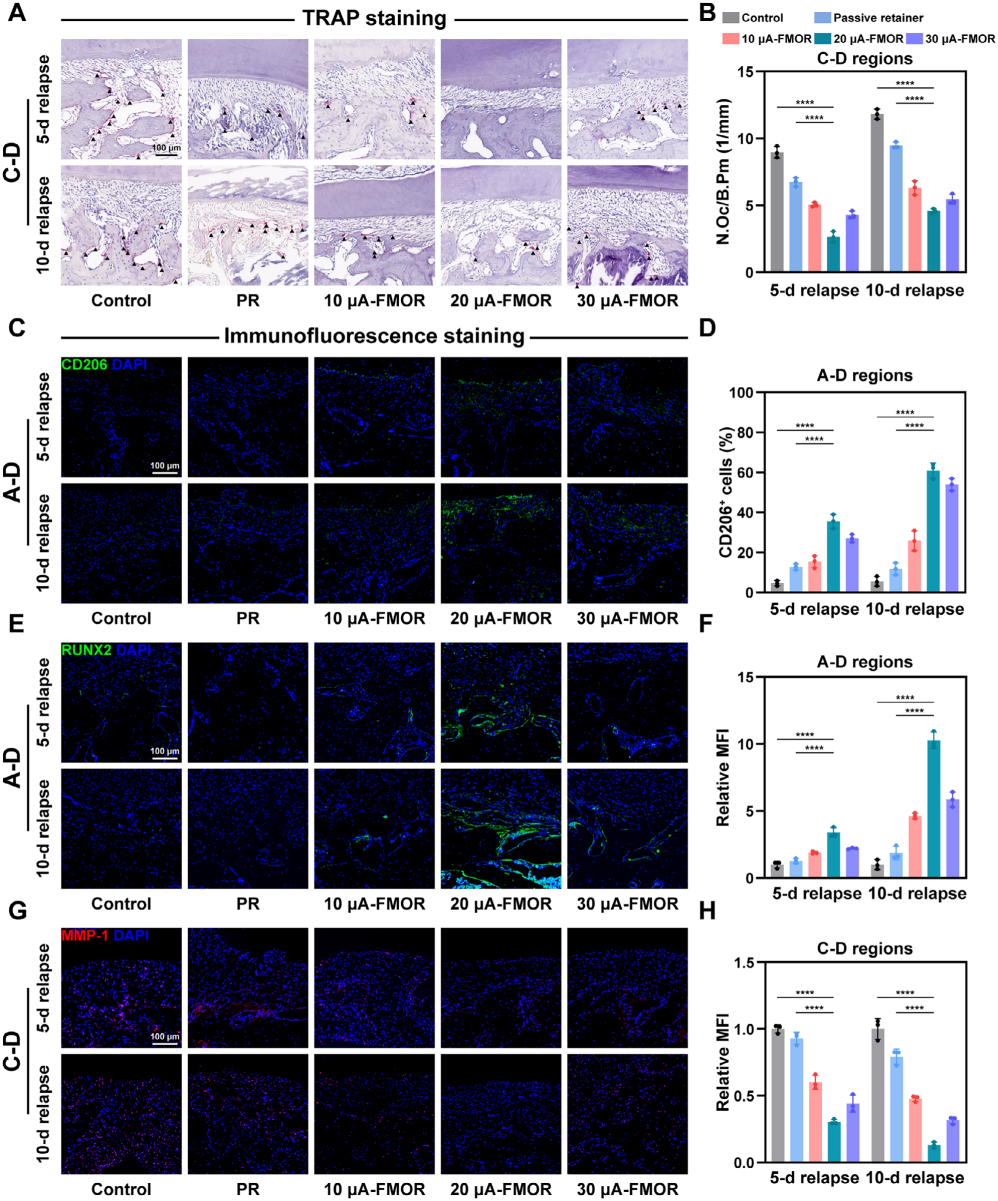
Magnetoelectric output remodels periodontal microenvironment in orthodontic relapse. A) TRAP staining on histological sections of alveolar bone resorption in C‐D region. Black triangles indicate TRAP‐positive cells. B) Quantitative analysis of the number of osteoclasts. C,D) Representative immunofluorescence images and quantitative analysis of CD206 in histological sections of alveolar bone resorption in A‐D region. E,F) Representative immunofluorescence images and quantitative analysis of RUNX2 in histological sections of alveolar bone generation in A‐D region. G,H) Representative immunofluorescence images and quantitative analysis of MMP‐1 in histological sections of periodontal membrane resorption in C‐D region. *p < 0.05, **p < 0.01, ***p < 0.001 and ****p <0.0001. Sample size (n = 3).

To further investigate the role of magnetoelectric retention therapy during the relapse phase, we performed tissue staining on rat maxillary specimens after 5 and 10 days of retention. TRAP and Cathepsin K (CTSK) are relevant indicators that respond to osteoclastic activity and bone resorption as previously mentioned.^[^ ^51^ ^]^ The results of the TRAP staining and osteoclast density analysis demonstrated that 20 µA‐FMOR therapy markedly suppressed osteoblastic differentiation on the bone resorption side (C‐D region) in comparison to the control and PR groups (**Figure** **4**A,B). Concurrently, on the bone deposition side (A‐D region), magnetoelectric retention therapy also notably expedited the clearance of TRAP^+^ osteoclasts generated during the OTM phase (Figure S9, Supporting Information). Immunofluorescence staining and intensity quantification of CTSK in C‐D and A‐D regions reconfirmed the inhibition of osteoclastic activity, which was consistent with the TRAP results (Figure S10, Supporting Information).

To further assess the role of magnetoelectric retention therapy on the reconstruction of the tissue microenvironment, the number of M2 macrophages were counted and analyzed.^[^ ^53^ ^]^ As mechano‐induced aseptic inflammation in orthodontics, shifts in macrophage M1/M2 polarization within the periodontal ligament (PDL) critically regulate inflammatory and regenerative processes.^[^ ^14^ ^]^ During early OTM, macrophages are recruited to inflammatory sites where cytokines drive predominant M1 polarization, which is a phenotype associated with sustained tissue destruction and root resorption. Conversely, M2 macrophages emerge significantly only during late OTM phase and after force removal, which orchestrate bone resorption cessation and initiate tissue repair. Crucially, prior studies confirm that elevating M2 proportions accelerates post‐inflammatory periodontal regeneration,^[^ ^54^ ^]^ while prolonged M1 dominance exacerbates damage.^[^ ^53^ ^]^ Immunofluorescence staining for CD206 showed that 20 µA‐FMOR therapy promoted sustained M2 macrophage polarization in both C‐D and A‐D regions, achieving inhibition of osteoclastic behavior in the bone resorption areas as well as promoting osteogenesis and tissue repair in the bone deposition areas (Figure 4C,D and Figure S11, Supporting Information).

Furthermore, to understand the role of FMOR on bone regeneration, runt‐related transcription factor 2 (RUNX2) were investigated.^[^ ^55^ ^]^ Immunofluorescence staining images showed that RUNX2 was abundantly expressed in the alveolar bone in A‐D region under 20 µA‐FMOR therapy, suggesting that magnetoelectric cues have a facilitating effect on osteogenesis (Figure 4E,F). Meanwhile, compared with the control and PR groups, 20 µA‐FMOR therapy similarly promoted an increased number of RUNX2^+^ cells in the bone resorption zone (Figure S12, Supporting Information). Considering the simultaneous inhibition of osteoclastic behavior in the C‐D region by magnetoelectric retention therapy, this suggests that magnetoelectric cues help to rebalance osteoblast and osteoclast activity, thereby promoting local bone repair and counteracting pressure‐induced bone resorption behavior. Finally, to explore the effect of magnetoelectric retention therapy on periodontal fiber remodeling, matrix metalloproteinase‐1 (MMP‐1) was labelled as an important protease for extracellular matrix and collagen fiber resorption.^[^ ^56^ ^]^ The results showed that 20 µA‐FMOR therapy significantly reduced the intensity of MMP1 expression in the periodontal membranes of C‐D and A‐D regions, which effectively protected the periodontal fibers on the pressure side from resorption and promoted matrix fiber restoration on the tension side (Figure 4G,H and Figure S13, Supporting Information).

In summary, due to aseptic inflammation caused by orthodontic force during OTM phase and pressure‐induced resorption of the unbalanced periodontal tissues during the relapse phase, self‐reconstruction of periodontal tissues is slowly initiated. By promoting matrix collagen metabolism and deposition, M2 macrophage polarization, rebalancing of osteogenesis and osteoclasis, magnetoelectric retention therapy thus dominate the reversal of periodontal destruction during the relapse phase.

### 2.5 Magnetoelectric Output Mediates Immune Remodel and Initiates Restorative Activity After Removal of Orthodontic Pressure In Vitro

**Figure 5:**
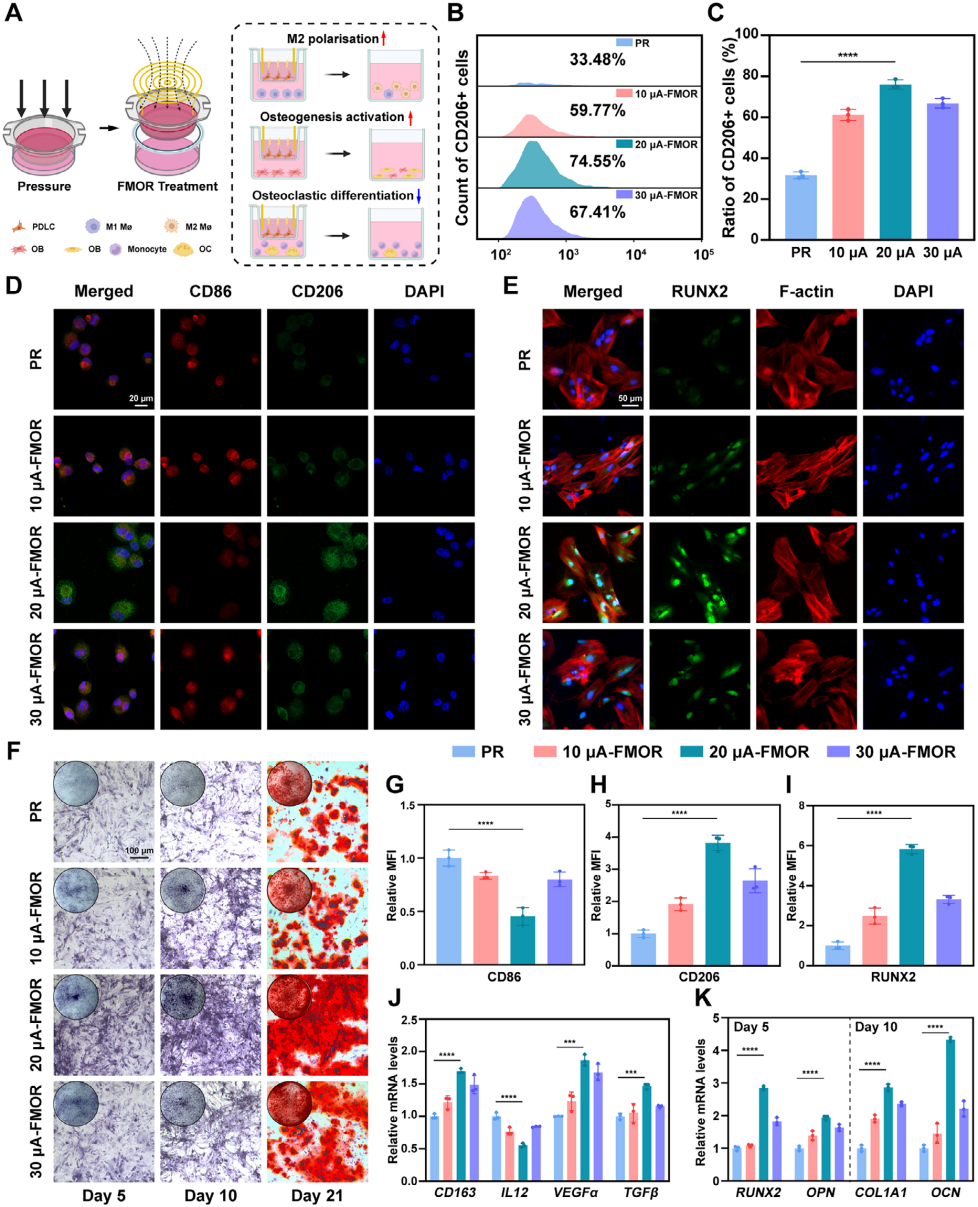
Magnetoelectric output mediates immune remodeling and promotes alveolar bone osteogenesis in vitro. A) Schematic diagram of the Transwell co‐culture system of PDLCs with other cells under wireless magnetoelectric output. Created by BioRender.com. B,C) Flow cytometry and quantitative analysis of the cell surface marker CD206 in macrophages co‐cultured with PDLCs under wireless magnetoelectric output. D,G,H) Representative immunofluorescence images and quantitative analysis of CD206 (green), CD86 (red) and cell nuclei (DAPI, blue) in macrophages co‐cultured with PDLCs under wireless magnetoelectric output for 48 h. E,I) Representative immunofluorescence images and quantitative analysis of RUNX2 (green), F‐actin (red) and cell nuclei (DAPI, blue) in h‐FOBs co‐cultured with PDLCs under wireless magnetoelectric output for 5 days. F) Representative images of ALP staining and Alizarin Red staining of h‐FOBs co‐cultured with PDLCs under wireless magnetoelectric output for 5,10, and 21 days. J) mRNA expression levels of representative cytokine genes in macrophages. K) mRNA expression levels of classic osteogenic gene markers in h‐FOBs for 5 and 10 days. *p < 0.05, **p < 0.01, ***p < 0.001 and ****p < 0.0001. Sample size (n = 3).

To clarify the intrinsic connection between magnetoelectric retention therapy and periodontal remodel, cell co‐culture system was used to simulate the periodontal microenvironment in vitro (**Figure** **5**A). During orthodontic treatment, periodontal membrane cells (PDLC) function as the primary sensors in response to mechanical signals that not only regulate periodontal soft tissue remodeling but also rely on the paracrine signaling network to control the local immune microenvironment and alveolar bone remodeling.^[^ ^57^ ^]^ Based on this, primary human PDLCs were seeded in Transwell chambers and applied a pressure of 2 g cm^−2^ for 24 h to mimic the in vivo orthodontic pressure during OTM phase as previously described.^[^ ^50^ ^]^ Subsequently, following magnetoelectric retention therapy for PDLC, the Transwell chambers were transferred to culture plates inoculated with macrophages, osteoblasts or osteoclasts, respectively, for cell co‐culture. Quantification of CD206^+^ macrophages by flow cytometry showed a massive increase in CD206^+^ macrophages from 33.48% to 74.55% in 20 µA‐FMOR therapy group compared with the control group (Figure 5B,C). Meanwhile, the results of immunofluorescence staining of macrophages demonstrated that magnetoelectric retention therapy markedly reduced the expression of the M1 macrophage marker CD86 while concurrently elevating the expression of the M2 macrophage marker CD206(Figure 5D,G,H). In addition, real‐time quantitative PCR (RT‐qPCR) results showed that the expression of *IL12*, a gene associated with M1 polarization, was significantly decreased in macrophages in 20 µA‐FMOR therapy group, whereas the expression of the genes *CD163*, *VEGFα*, and *TGFβ*, associated with M2 polarization, was significantly increased (Figure 5J).

**Figure 6:**
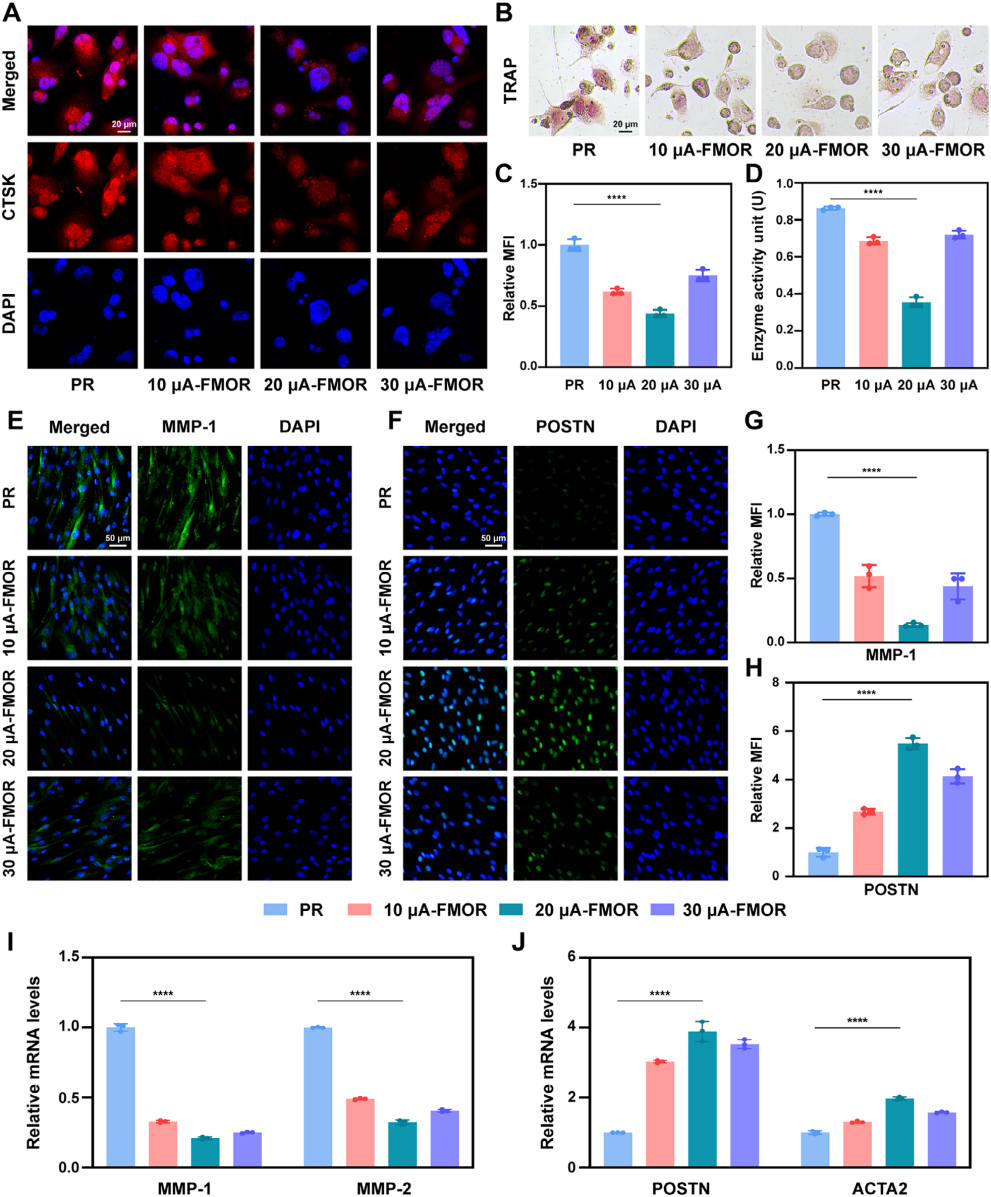
Magnetoelectric output inhibits osteoclastic behavior and promotes periodontal remodeling in vitro. A,C) Representative immunofluorescence images and quantitative analysis of CTSK (red) and cell nuclei (DAPI, blue) in OCs co‐cultured with PDLCs under wireless magnetoelectric output for 10 days. B,D) Representative images of TRAP staining and the TRAP activity of OCs co‐cultured with PDLCs under wireless magnetoelectric output for 10 days. E,F,G,H) Representative immunofluorescence images and quantitative analysis of MMP‐1 (green), POSTN (green) and cell nuclei (DAPI, blue) of PDLCs under wireless magnetoelectric output for 10 days. I) mRNA expression levels of representative genes associated with fiber and extracellular matrix disruption in PDLCs for 10 days. J) mRNA expression levels of representative genes associated with fiber and extracellular matrix reconstruction in PDLCs for 10 days. *p < 0.05, **p < 0.01, ***p < 0.001 and ****p < 0.0001. Sample size (n = 3).

To explore the balance of bone deposition resorption with magnetoelectric retention therapy, osteogenesis and osteoclasis was evaluated similarly in vitro. Immunofluorescence of human osteoblasts (hFOBs) showed that RUNX2 (Figure 5E,I) and COL1A1 (Figure S14, Supporting Information) exhibited the highest expression intensity with 20 µA‐FMOR therapy. Alkaline phosphatase (ALP) staining at 5 and 10 days was conducted to demonstrate the osteogenesis process of hFOBs in a cell co‐culture system. Additionally, Alizarin Red staining was employed to label the extracellular calcified nodules after 21 days. The results indicated that the 20 µA‐FMOR therapy exhibited the highest osteogenic and calcification efficiencies (Figure 5F). Moreover, the expression of osteogenic‐related marker genes *RUNX2*, *OPN*, *COL1A1* and *OCN* were analyzed by RT‐qPCR after 5‐ and 10‐day culture, suggesting 20 µA‐FMOR therapy provided the highest osteogenic efficiency (Figure 5K). On the other hand, immunofluorescence staining of human osteoclasts showed that 20 µA‐FMOR therapy significantly downregulated CTSK expression (**Figure** **6**A,C). TRAP staining and quantitative measurement confirmed that 20 µA‐FMOR therapy significantly downregulated TRAP activity by more than half compared to the control group. (Figure 6B,D).

Last, the effect of magnetoelectric retention therapy on periodontal fibre metabolism was assessed. Immunofluorescence staining of PDLCs showed that MMP‐1 associated with collagen fiber resorption was significantly down‐regulated with 20 µA‐FMOR therapy (Figure 6E,G). Whereas the periostin (POSTN), which is associated with PDLCs proliferation as well as periodontal fiber secretion and reconstruction,^[^ ^58^ ^]^ was significantly up‐regulated (Figure 6F,H). Gene expression levels related to extracellular matrix and collagen metabolism were examined by RT‐qPCR. Results showed that *MMP‐1* and *MMP‐2* were significantly down‐regulated (Figure 6I), while *POSTN* and *ACTA2* was increased in PDLCs with 20 µA‐FMOR therapy (Figure 6J), suggesting deposition and remodeling of extracellular matrix and fibrillar collagen.

### 2.6 Magnetoelectric Output Remodels the Periodontal Microenvironment via Modulating the Immune Signaling and Metabolic Pattern of PDLCs

**Figure 7:**
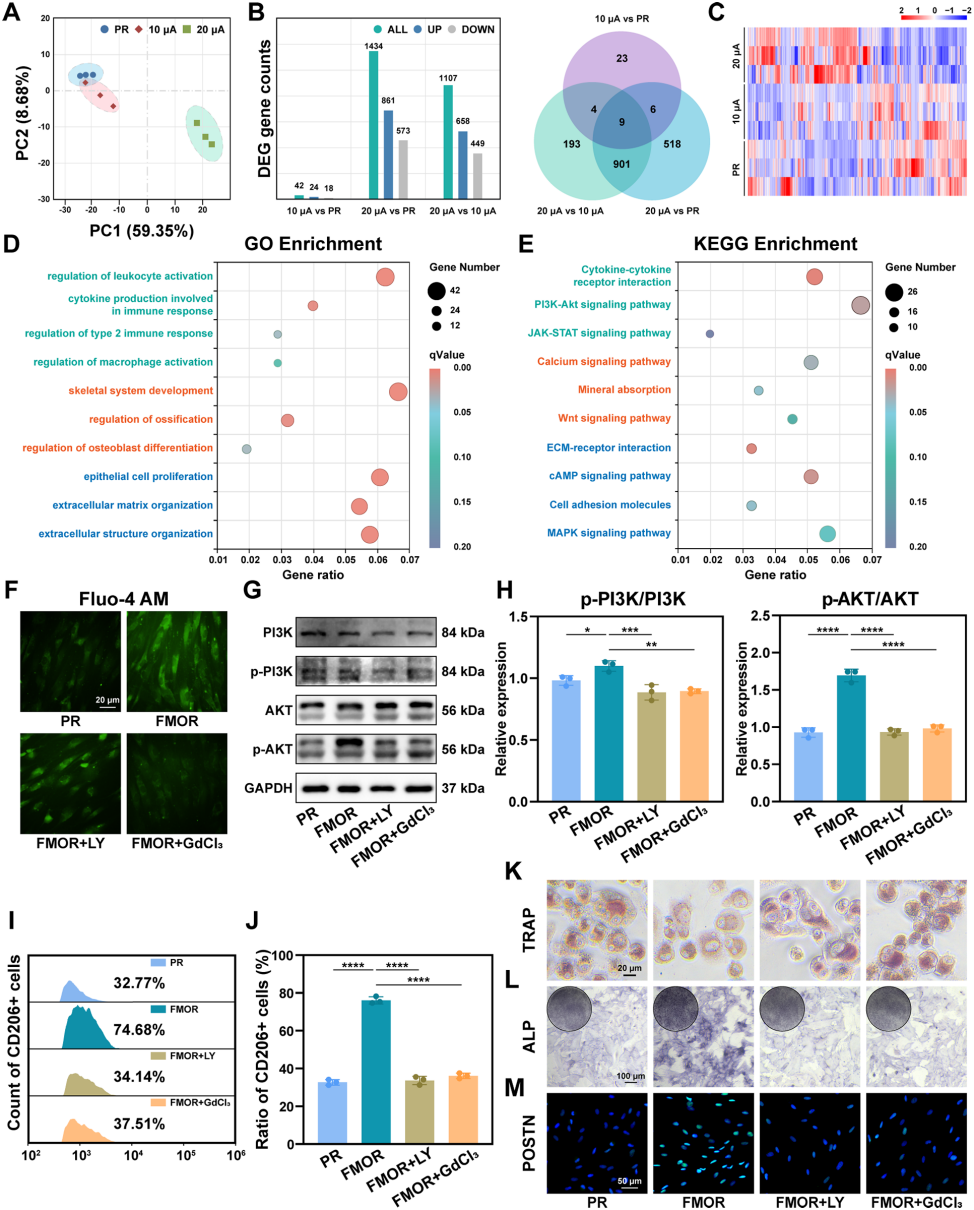
Magnetoelectric output modulates the immune signaling and metabolic patterns of PDLCs. A) Principal Component Analysis (PCA) of the RNA sequencing profile of PDLCs after 7 days of culture. B) Differentially expressed genes from RNA‐seq analysis of PDLCs after 7 days of culture. C) The heatmap of differentially expressed genes from RNA sequencing. D) Gene Ontology (GO) enrichment analysis of the upregulated genes in PDLCs cultured after 7 days. E) Kyoto Encyclopedia of Genes and Genomes (KEGG) analysis of upregulated genes in PDLCs cultured after 7 days. F) Representative immunofluorescence images of Flou‐4 (green) signals in PDLCs cultured for 7 days. G,H) Western blot images and quantitative analysis of the phosphorylation levels of PI3K and AKT in PDLCs in different groups. I,J) Flow cytometry and quantitative analysis of the cell surface marker CD206 in macrophages co‐cultured with PDLCs in different groups. K) Representative images of TRAP staining of OCs co‐cultured with PDLCs in different groups for 10 days. L) Representative images of ALP staining of h‐FOBs co‐cultured with PDLCs in different groups for 5 days. M) Representative immunofluorescence images POSTN (green) and cell nuclei (DAPI, blue) of PDLCs in different groups for 10 days. *p < 0.05, **p < 0.01, ***p < 0.001 and ****p < 0.0001. Sample size (n = 3).

To further elucidate the response of the periodontal microenvironment to the magnetoelectric retention therapy from transcriptomic perspective, we performed RNA sequencing on PDLCs after 7‐day magnetoelectric retention therapy in vitro. Principal component analysis (PCA) demonstrated a notable divergence in global gene expression in 20 µA‐FMOR group (**Figure** **7**A). Differential Expression Gene (DEG) counts and Wayne plots among groups showed that, compared with the other two group, the number of DEGs up‐regulated in the 20 µA‐FMOR group was 1434 and 1107, respectively, of which 910 DEGs were overlapped (Figure 7B,C). These results provided genetic validation of the previous results, indicating that a significant effect of magnetoelectric retention therapy need the magnetoelectric output to reached 20 µA.

Further, GO enrichment analysis revealed that the up‐regulated DEGs are mainly related to macrophage activation, type 2 immune response, epithelial cell proliferation, extracellular matrix metabolism, and skeletal system development in 20 µA‐FMOR group (Figure 7D). Furthermore, KEGG pathway enrichment analysis indicated that the upregulation of PI3K‐Akt signaling pathway, Calcium signaling pathway, cAMP signaling pathway, MAPK signaling pathway and etc. was correlated with 20 µA‐FMOR therapy (Figure 7E). Previous studies have investigated that activated PI3K‐Akt signaling pathway demonstrates superiority in inducing PDLCs to immune repair, collagen formation and promote alveolar bone mineralization by up‐regulating TGFβ and M2‐type macrophage polarization,^[^ ^59^, ^60^ ^]^which potentially linked to orthodontic relapse inhibition.^[^ ^61^ ^]^ Meanwhile, Established evidence positions calcium ions (Ca^2^⁺) as critical secondary messengers in intracellular signaling, with growing literature emphasizing electro‐activated Ca^2^⁺ influx in triggering downstream pathways.^[^ ^62^ ^]^

Based on this, immunohistochemical staining of key marker proteins (p‐PI3K, PI3K, p‐AKT, AKT) in PI3K‐AKT signaling pathway was performed in rat periodontal specimens (Figure S15, Supporting Information). The results of the phosphorylation ratios preliminarily confirmed the effect of magnetoelectric retention therapy on the activation of PI3K‐AKT signaling pathway in vivo. Subsequently, in vitro rescue experiments were designed to further investigate the signaling between the pathways. To validate the roles of calcium signaling and PI3K‐AKT pathways, we introduced gadolinium chloride (GdCl₃) to inhibit calcium channels in FMOR group (FMOR+GdCl₃ group) and LY294002 (FMOR+LY group) to suppress PI3K‐AKT activation. Fluo‐4 AM fluorescence imaging revealed significantly elevated intracellular Ca^2^⁺ levels in FMOR group versus PR control, while FMOR+GdCl₃ group showed no significant difference from PR levels (Figure 7F). This result confirmed that magnetoelectric retention therapy activated calcium signaling to enhance intracellular Ca^2^⁺ concentration. Western blot analysis further demonstrated that calcium channel activation initiates downstream PI3K/AKT signaling. Phosphorylation levels of PI3K/AKT pathway proteins were markedly higher in FMOR group than PR control, consistent with the RNA sequencing data (Figure 7G,H). Notley, both GdCl₃ and LY294002 reduced phosphorylation to PR baseline levels. Subsequently, in vitro functional assays including macrophage polarization (flow cytometry of CD206 positive macrophages in Figure 7I,J), osteogenic/osteoclastic activity (ALP staining of h‐FOBs in Figure 7L and TRAP staining of osteoclasts in Figure 7K), and collagen formation (immunofluorescence images of POSTN of PDLCs in Figure 7M) revealed that GdCl₃ treatment abolished FMOR therapeutic effects, comparable to that of the FMOR+LY group.

Collectively, these results suggested a coherent mechanistic framework wherein magnetoelectric retention therapy may involve Ca^2^⁺ influx through PDLC calcium channels. In this model, Ca^2^⁺ functions as a secondary messenger, potentially activating PI3K/AKT signaling during magnetoelectric retention therapy. This cascade could initiate paracrine signaling networks that contribute to periodontal reconstruction and relapse inhibition. Notably, considering that ion signaling is also associated with mechanotransduction channels, such as Piezo1, the above mechanisms require additional validation beyond KEGG analysis.

### 2.7 Application of FMOR Greatly Inhibits Post‐Orthodontic Relapses in a Rabbit Model

**Figure 8:**
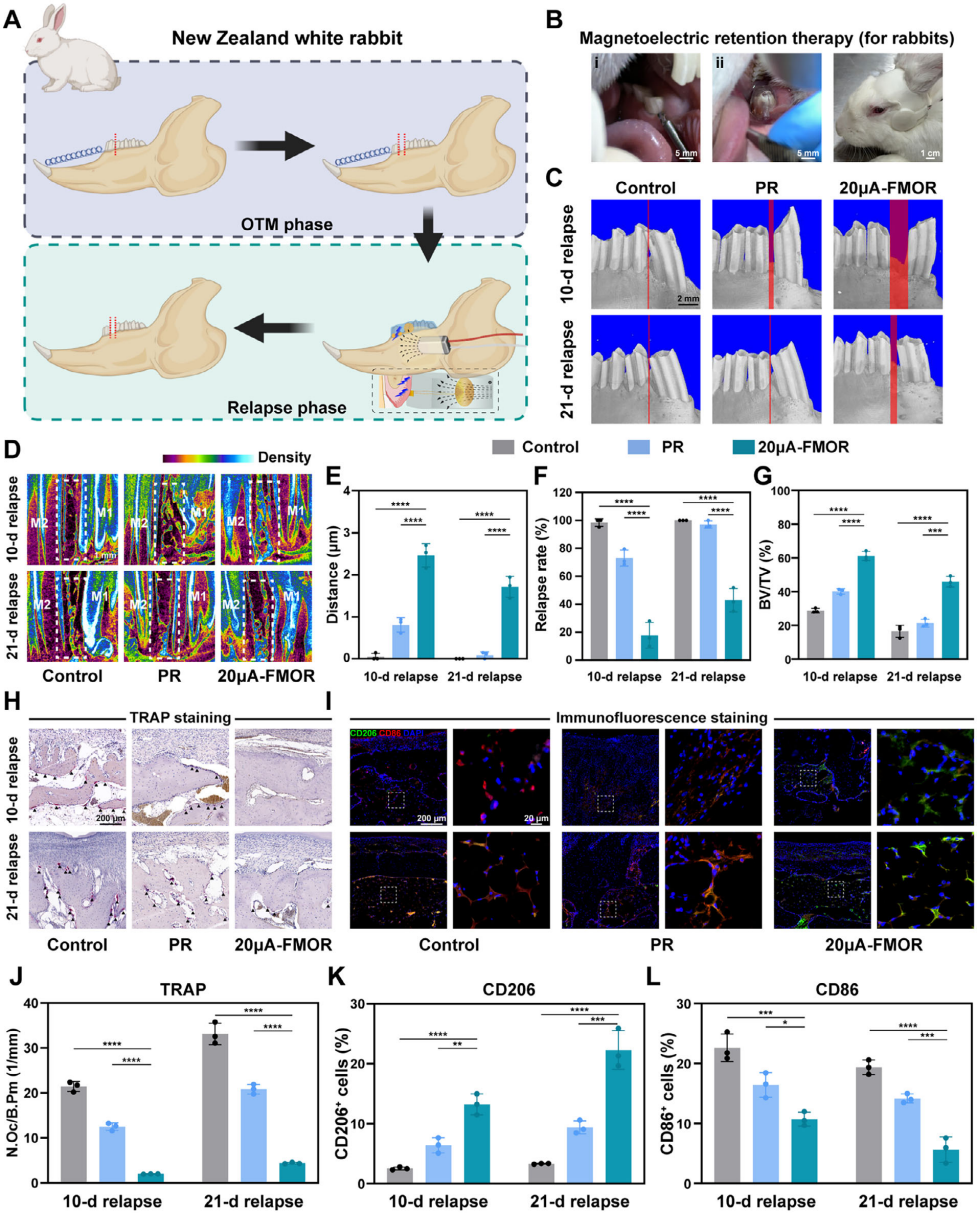
FMOR magnetoelectric retention therapy greatly improved orthodontic retention efficiency in vivo. A) Schematic illustration of the orthodontic and relapse rabbit model. Created by BioRender.com. B) Magnetoelectric retention therapy for rabbits in vivo: i) Moving the first molar using springs with a force of 100 g in the OTM phase. ii) Wearing personalized FMORs for each rabbit. iii) Initiating magnetoelectric retention therapy with the wearable magnetoelectric transmitter. C) Representative micro‐CT images of rabbit dentition after 10 and 21 days of orthodontic relapse. The width of the red area indicates the distance between the first and second molars. Scale bar = 1 mm D) Micro–computed tomography images of alveolar bone between M1 and M2 in rabbits. E,F) Quantitative analysis of the distance between first and second molars and the relapse rate. Relapse rate = relapse distance / relapse distance at 0 d. G) Quantitative analysis of percent bone volume (BV/TV) of alveolar bone between M1 and M2 in rabbits. H,J) TRAP staining and quantitative analysis of the number of osteoclasts on histological sections of alveolar bone resorption regions. Black triangles indicate TRAP‐positive cells. I,K,L) Representative immunofluorescence images and quantitative analysis of CD206 (green) and CD86 (red) in histological sections of alveolar bone resorption regions. *p < 0.05, **p < 0.01, ***p < 0.001 and ****p < 0.0001. Sample size (n = 3).

Considering the constrained jaw dimensions, a 3‐week OTM phase, and an applied force of 40 grams in the rat model, it is slightly less appropriate for assessing magnetoelectric retention therapy under clinically relevant orthodontic forces (i.e., ≥50 grams) or extended OTM distances. In contrast, the rabbit model exhibited a higher tolerance for orthodontic forces (100 g) and a greater OTM distance (six times the distance observed in the rat model).^[^ ^63^ ^]^ This greater capacity enables a better simulation of clinical situations of periodontal remodeling subsequent to tooth movement (**Figure** **8**A). As described previously, the mandibular first molars of the rabbit entered the relapse phase after rapidly moved by a constant orthodontic force of 100 g.^[^ ^64^ ^]^ During the relapse phase, rabbits were treated with magnetoelectric retention therapy for 12 h (Figure 8B). After 10 and 21 days of relapse, micro‐CT imaging demonstrated that the 20 µA‐FMOR therapy exhibited the smallest relapse distance (Figure 8C,E), while effectively reducing the 100% relapse rate observed in the control and PR groups to ≈40% for 21 days (Figure 8F). Representative tomographic reconstruction images and bone volume fraction results showed that 20 µA‐FMOR therapy was effective in promoting bone repair and increasing alveolar bone density in relapse phase (Figure 8D,G). The number of TRAP‐positive multinucleated cells reduced significantly in the 20 µA‐FMOR therapy group, indicating an effective inhibition in osteoclast differentiation during relapse phase (Figure 8H,J). Furthermore, immunofluorescence staining of alveolar bone showed a decrease of CD86^+^ and an increase of CD206^+^ macrophages with 20 µA‐FMOR therapy (Figure 8I,K,L), confirming the magnetoelectric retention therapy remodeled periodontal microenvironment by promoting M2‐type macrophage polarization in relapse phase.

Therefore, compared with the control and PR groups in the rabbit model, we further identified the efficacy and clinical prospects of FMOR. Due to the lack of physiological active regulation, the retention effect of the clinical passive retainer was limited for its restricted wearing time per day. However, FMOR achieves a significant retention efficiency for its periodontal immune environment reversal, osteogenesis and osteoclastic balance and periodontal fiber reconstruction. In conclusion, the development of FMOR with active regulation is of clinical significance and provides insights for optimizing orthodontic relapse treatment.

## 3 Conclusion

In conclusion, the magnetoelectric retention therapy has been successfully developed for efficient periodontal restoration and reconstruction after orthodontics. The therapy consists of an additively fabricated flexible magnetoelectric orthodontic retainer (FMOR) configured with a receiving liquid metal (LM) coil and a wearable magnetoelectric transmitter configured with a transmitting LM coil. Following orthodontic treatment, the patient wears the FMOR, which generates a self‐powered magnetoelectric output through wireless remote modulation of the wearable magnetoelectric transmitter. Thus, magnetoelectric cues reversed pressure‐induced aseptic inflammation and promoted reconstruction of the periodontal environment, which triggers M2 macrophage polarization, rebalances osteoclastic and osteoblastic activity and facilitates collagen deposition. The mechanistic exploration revealed that 20 µA‐FMOR therapy significantly upregulated the genes related to immune response, extracellular matrix organization, and osteogenesis regulation in PDLC. Our study provides a high‐performance strategy for optimizing orthodontic retention therapy, highlighting the promise of additively fabricated flexible magnetoelectric devices for clinical tissue repair and reconstruction.

## 4 Experimental Section

### Fabrication of the FMOR system

1) Preparation and encapsulation of FMOR

The maxilla of the rats and mandible of the rabbits were recorded into digital models using a 3D scanner (Creality 3D CR‐Scan Otter), which preserves various detailed features of teeth. The results of the scanning were designed into braces that fit the teeth using model design software, and the corresponding detachable negative and positive molds were designed. The mold parts were then printed and shaped using a commercial light‐cured printing device (Formlabs Form3+). In order to allow FMOR to be smoothly peeled off from the molds, the residual photosensitive resin on the surface of the molds was removed here using a plasma cleaner (PD150).

The base polymer of polydimethylsiloxane (PDMS) was mixed with the cross‐linking agent in a mass ratio of 10:1, stirred thoroughly and vacuumed to make the precursor liquid. The prepared precursor liquid was injected into the mold in layers, and a prefabricated copper coil and its output electrode with a layered spatial structure were placed. The FMOR was crosslinked at a temperature of 60 °C for 2 h. FMOR was prepared by removing the PDMS from the mold after curing.

2) Preparation and encapsulation of the wearable magnetoelectric transmitter

Head structures of rats and rabbits were recorded as digital models using a 3D scanner. Model design software was used to create a model of a headgear universally suitable for rats or rabbits with a circular slot in the cheek area directly opposite the teeth. The headgear was printed using a light‐curing printing device. Electronic components such as the control chip, voltage regulator module, rectifier module, and communication module were integrated into the main board and placed in the circular slot together with the emitting copper coil. The wearable magnetoelectric transmitting coil is powered by a dry battery and outputs magnetoelectric signals to the FMOR.

### General Characterization of the FMOR System

1) Structural characterization

To evaluate the mechanical behavior of the PDMS, we prepared the precursor solution using a consistent mixing ratio. Two types of 1 cm^3^ PDMS solid blocks — one with embedded coils and one without — were fabricated using a mold. Stress‐strain data were obtained under uniaxial compression with a displacement of 1 mm. Young's modulus was calculated using the formula:

(3) $$E=\frac{\sigma }{\varepsilon }=-\frac{FL_{0}}{A\left(L-L_{0}\right)}$$

The FMOR was placed 5 cm away from the emitting coil and the emitting coil was kept at an output current of 50 µA. An infrared camera was used to measure the temperature change of the FMOR.

2) Electrical characterization and signal acquisition

In order not to interfere with the normal activities of the experimental subjects, the orthodontic retention system was designed as two parts, the transmitting end and the receiving end. The receiving end is fixed to the rat's teeth by FMOR, and two electrode sheets are affixed to the area to be electrically stimulated. The transmitting end converts the direct current from the dry battery into an alternating current that changes periodically, and the alternating magnetic field formed supplies energy to the receiving end through electromagnetic induction. The electrical stimulation waveform of the electrodes at the receiving end can be changed by modulating the waveform characteristics at the transmitting end.

In order to investigate the ability of the retainer to cope with various states during use, the experiment was conducted by modulating the relevant variables to simulate different scenarios. In actual use, the coils at the transmitter and receiver ends do not guarantee an absolute positional relationship. In the experiment, the horizontal height distance, axial central distance, relative angle θ and input voltage of the two coils are adjusted to investigate the transformation of the stimulus current at the output end under different states.

### Animals

All animals were purchased from Beijing Vital River Laboratory Animal Technology Company. All animal experiments were authorized by the Animal Ethics Committee of Tongji Medical College (Wuhan, China) and approved by the Institutional Animal Care and Use Committee of Tongji Medical College ([2022] IACUC Number: 3977).

Male 6‐week‐old Sprague‐Dawley (SD) rats were randomly divided into five groups. The rats were housed under specific pathogen free (SPF) conditions with standard rodent chow and provided free access to water during a 12‐h light‐dark cycle. After 2 weeks of acclimatization, the rats were anaesthetized with an intraperitoneal injection of sodium pentobarbital (40 mg kg^−1^ body weight) and a 0.2 mm nickel‐titanium helical spring, adjusted to a tensile force of ≈40 g, was attached between the maxillary first molar and the maxillary incisor. In order to minimize the effects of mesial tooth displacement, the orthodontic appliance was checked daily and also reactivated weekly. After 3 weeks of loading and the M1 reaches an OTM distance of 500 µm, the orthodontic appliance was removed and the rats entered the relapse phase. The rats were treated with magnetoelectric retention therapy during the relapse phase. Specifically, rats were fitted with FMOR for a period of 12 h each day. The wearable magnetoelectric transmitter device was adjusted to set varying magnetoelectric output gradients (10, 20, and 30 µA). After 5 and 10 days of relapse, Micro‐CT 3D remodeling and sagittal images of rat maxillary molars was performed and the distance between M1 and M2 was measured.

Similarly, male 2.5 kg New Zealand rabbits were randomized into 3 groups. After 2 weeks of adaptation to the environment, the rabbits were anaesthetized with an intravenous injection of 2% sodium pentobarbital saline solution (1 mL kg^−1^ body weight) at the ear margin, and a 0.2‐mm nickel‐titanium coil spring, adjusted to a tension of ≈100 g, was attached between the mandibular first molar and the maxillary incisor. Meanwhile, the orthodontic appliance was checked daily while it was reactivated weekly. After loading for 4 weeks and the M1 reaches an OTM distance of 3 mm, the orthodontic device was removed and the rabbits entered the relapse phase. The rabbits were treated with magnetoelectric retention therapy during the relapse phase. Specifically, the rabbits were treated with magnetoelectric retention therapy by wearing FMOR for 12 h per day. The wearable magnetoelectric transmitters were adjusted to set magnetoelectric output intensities of 20 µA. FMOR group was compared with PR group (without initiating magnetoelectric retention treatment) and Control group (without wearing FMOR). After 10 and 21 days of relapse, Micro‐CT 3D remodeling was conducted and specimens were harvested for histological analysis.

### Micro‐CT Scanning Evaluation

Animal intraoral measurements were obtained through standardized micro‐CT scanning (SkyScan 1176, Bruker, Belgium) followed by 3D reconstruction and quantitative analysis. Specifically, animals were scanned at 70 kV, 350 µA, 180° rotation, 0.3° rotation step and 9 µm resolution after survival anesthesia (OTM phase) or terminal anesthesia (relapse phase). Raw datasets were reconstructed using NRecon (v1.6.10.4, Bruker) then analyzed in DataViewer (v1.5.2.4, Bruker) and CTAn (v1.15.4.0, Bruker) with triple measurements per parameter.

With reference to other relevant studies,^[^ ^65^ ^]^ the OTM distance was defined as the distance between the nearest contact points of the first molar (M1) and the second molar (M2) at the end of the OTM phase. Relapse distance was the OTM distance minus the distance between the nearest contact points of M1 and M2 after relapse. The relapse rate is the ratio of the relapse distance to the OTM distance. Each result was measured three times.

### Hematoxylin Eosin Staining

Maxillary specimens were decalcified with 10% ethylenediaminetetraacetic acid (EDTA) decalcification solution after micro‐CT scanning. When the bone tissue was softened, the samples were observed morphologically following a routine Hematoxylin Eosin (H&E) staining procedure. Briefly, the samples were dehydrated, embedded in paraffin and cut into 4 µm thick sections. After deparaffinization and rehydration, the sections were stained with hematoxylin and differentiated with acidic alcohol. Subsequently, the specimen sections were stained with eosin solution, then dehydrated and cleared. Histological images were taken with an optical microscope.

### TRAP Staining

The paraffin sections were prepared as described above. TRAP staining was performed according to the instructions of the TRAP kit (Solarbio, China) to label osteoclasts. Briefly, TRAP incubation solution was prepared by mixing AS‐BI buffer, GBC solution and TRAP buffer in the ratio of 10:1:90. After deparaffinization and rehydration, the sections were rinsed with TRAP Incubation Solution for 45–60 min at 37 °C and then washed with ultrapure water. Sections were then restained with hematoxylin solution for 5 min. Relative images were obtained using an optical microscope.

### Immunofluorescence for Tissue Staining

Paraffin‐embedded maxillary and mandibular tissue sections were blocked with 5% bovine serum albumin (BSA) for 1 h to avoid non‐specific staining. Subsequently, the sections were incubated with primary antibodies CD206, CD86, RUNX2, MMP‐1 and CTSK at 4 °C overnight in a lucifugal chamber. Primary antibody dilutions were 1:200. The slides were then washed with PBS for three times, followed by incubation with secondary antibody (1:200) for 1 h in the dark. Cell nuclei were stained with 4′,6‐Diamidino‐2‐phenylin‐dole (DAPI, Sigma, USA) for 5 min. The fluorescent images were captured with a confocal microscope (Nikon A1‐Si, Japan). The antibodies used in immunofluorescence are described in Table S2, Supporting Information.

### Cell Culture

Human periodontal cells (PDLCs) were obtained from orthodontically extracted premolar teeth. Briefly, the periodontal ligaments of premolar teeth were collected with a sterile blade, followed by digestion of the periodontal ligaments with collagenase I (C8140, Solarbio) at 37 °C for 1 h. PDLCs were cultured in T25 flasks with α‐MEM medium containing 10% fetal bovine serum (FBS, Gibco, USA) and 1% penicillin‐streptomycin (HyClone, USA), which was incubated in a humidified incubator containing 5% CO_2_ at 37 °C. PDLCs were passaged every 5 days and were used in this research were in passages 4–6.

The human monocytic leukemia cell line (THP‐1) was obtained from the American Type Culture Collection (ATCC). THP‐1 cells were maintained in RPMI 1640 medium (HyClone, USA) supplemented with 10% FBS and 1% penicillin–streptomycin in an incubator at 37 °C with 5% CO_2_. THP‐1 cells were differentiated in different ways to obtain macrophages or osteoclasts. With 100 ng mL^−1^ phorbol 12‐myristate 13‐acetate (PMA, P8139‐1MG, Sigma, USA) for 48 h, THP‐1 cells were differentiated into macrophages to probe the direction of macrophage polarization. To probe osteoclastic behavior, THP‐1 cells were differentiated into osteoblasts with 20 ng mL^−1^ receptor activator of nuclear factor κ‑B ligand (RANKL, Novoprotein, China), 20 ng mL^−1^ macrophage colony‑stimulating factor (M‐CSF, Novoprotein, China) and 100 ng mL^−1^ PMA for at least 10 days.

The human fetal osteoblast cell line (hFOB 1.19) was obtained from ATCC. The cells were cultured in DMEM/F12 medium (HyClone, USA) supplementing 0.3 mg mL^−1^ G418 (Biosharp, China), 10% FBS and 1% penicillin‐streptomycin. To evaluate osteogenic behavior, hFOB cells were activated osteogenic expression with 10 mmol L^−1^ β‐glycerophosphate (Sigma, USA), 10 nmol L^−1^ dexamethasone and 50 µg mL^−1^ ascorbic acid for 7 days.

### Cell Co‐Culture System Simulating the Periodontal Microenvironment In Vitro

PDLCs were plated at a density of 1 × 10^5^ cells per well in Transwell chambers of 6‐well plates. When the cells were cultured to a density of 70% – 80%, they were continuously compressed according to the uniform compression method described previously.^[^ ^50^ ^]^ Briefly, the plates were placed in the Bionic Pressure Cell Culturator (NK‐P40, Naturethink, China) and the instrument parameters were adjusted to achieve a compressive force of 2 g cm^−2^ for 24 h in 5% CO_2_ at 37 °C. Next, the PDLC in the Transwell chambers were magnetoelectrically stimulated using a receiver coil, and the stimulation intensity was controlled by a transmitter coil. Finally, Transwell chambers were transferred to culture plates seeded with macrophages, osteoblasts or osteoclasts to mimic the state of the periodontal microenvironment during the relapse phase after orthodontic treatment.

### Flow Cytometry Analysis

Expression of the M2 macrophage surface marker CD206 was detected using flow cytometry. After 48 h of culture, cells were scraped, fixed with 4% (w/v) paraformaldehyde, blocked with 1% (w/v) BSA/PBS, and then incubated with FITC Anti‐Human CD206/MMR Antibody (E‐AB‐F1161C, Elabscience, China). Cells were analysed using a BD FACS Canto II flow cytometer.

### Alkaline Phosphatase and Alizarin Red S staining

Alkaline phosphatase assay was performed using the BCIP/NBT Alkaline Phosphatase Staining Kit (Beyotime, China). After 5 and 10 days of osteogenic activation of hFOB in an in vitro simulated periodontal microenvironmental cell co‐culture system, the medium was discarded and the cells were rinsed with PBS, and BCIP/NBT staining solution was added to each well (PBS, containing BCIP (3.3 µL mL^−1^) and NBT (6.5 µmol mL^−1^). Images were taken with a microscope after 30 min of incubation at room temperature.

Alizarin Red S Staining Kit (Beyotime, China) for displaying osteoblast mineralized nodules in vitro. As described previously, after 21 days of hFOB osteogenic activation, the culture medium was removed and fixed with 4% paraformaldehyde. After 3 washes using PBS, Alizarin Red S staining solution was added and stained for 30 min at room temperature. After a final thorough washing using distilled water, images were captured under a light microscope.

### Quantitative Real‐Time PCR

Total RNA was extracted using TRIZOL (TaKaRa, Japan) according to the manufacturer's instructions. Complementary DNA (cDNA) was reverse transcribed from the RNA templates using HiScript III RT SuperMix for qPCR (+gDNA wiper) (Vazyme, China). qRT‐PCR was performed using ChamQ Universal SYBR qPCR Master Mix (Vazyme, China) on Step One Plus real‐time PCR systems (Applied Biosystems, Thermo Fisher, USA). Quantification of target gene expressions was analyzed by the 2−ΔΔCt method, normalized to the expression of glyceraldehyde 3‐phosphate dehydrogenase (GAPDH) gene, and was presented as mean ± SD of replicates. The primer sequences used in qRT‐PCR are described in Table S3, Supporting Information.

### Immunofluorescence for Cell Staining

Cells were fixed with 4% paraformaldehyde for 15 min and then permeabilized by 0.5% Triton X‐100 for 15 min. Subsequently, cells were blocked with 5% bovine serum albumin (BSA) for 1 h at room temperature. The cells were incubated overnight at 4 °C in a humid chamber using primary antibody (1:200). Cells were washed three times the following day and incubated for 1 h at room temperature with secondary antibody. Cytoskeleton was stained with TRITC Phalloidin (YEASEN, China) for 30 min and cell nuclei were counterstained with DAPI for 5 min. Images were captured using a laser‐scanning confocal microscope (Nikon A1‐Si, Japan). The primary antibodies used in immunofluorescence are described in Table S2, Supporting Information.

### RNA Sequencing

PDLCs were treated with different intensities of magnetoelectric retention therapy for 7 days in the cell co‐culture system in vitro. Cell lysates were then collected using TRIZOL (TaKaRa, Japan) according to the manufacturer's instructions. Three independent replicate samples from three groups were sent to Beijing Novogene Co, Ltd. for global transcriptome analysis. Before transcriptome sequencing, quality control tests were performed on the samples, including 1) RNA concentration greater than 50 ng uL^−1^ and total amount greater than 1.5 µg; 2) 260 nm/280 nm absorbance ratio close to the range of 1.8–2.1, and the samples were free of macromolecular contamination; and 3) the samples were kept intact and free of degradation.

In the differential expression analysis, DESeq2 was applied to identify differentially expressed genes. Differentially expressed genes (DEGs) were chosen as *p*‐value < 0.05 and |FoldChange| ≥ 1. For Gene Ontology (GO) enrichment analysis of DEGs, clusterProfiler R package was implemented. GO terms with corrected p‐value <0.05 were considered significantly enriched by DEGs. The Kyoto Encyclopedia of Genes and Genomes (KEGG) enrichment analysis of differentially‐expressed genes was also performed by clusterProfiler R package. Differentially‐expressed genes were significantly enriched for KEGG pathways with a p‐value less than 0.05.

### Statistical Analysis

All numerical data were presented as mean ± standard deviation (SD) and statistically analyzed using GraphPad Prism 8.0 (GraphPad Software Inc., USA) with three or more replicate values. Significant differences were determined using Student's *t*‐test for comparison of two groups and one‐way ANOVA with post‐hoc Tukey's test for multiple comparisons. The statistical significance was declared when *p < 0.05, **p < 0.01, ***p < 0.001 and ****p < 0.0001.

### Ethics Approval and Consent to Participate

All animal experiments were performed in accordance with the Guidelines for Care and Use of Laboratory Animals of Tongji medical college, Huazhong University of Science and Technology and approved by the Animal Ethics Committee of Tongji medical college, Wuhan ([2022] IACUC Number: 3977).

## Conflict of Interest

The authors declare no conflict of interest.

## Author Contributions

H.L., J.S., and P.C. contributed equally to this work. H.L. contributed to writing – review & editing, writing – original draft, visualization, validation, methodology, investigation, formal analysis, data curation, and conceptualization; J.S. to writing – original draft, validation, investigation, funding acquisition, formal analysis, and conceptualization; P.C. to writing – original draft, investigation, formal analysis, and data curation; L.H. to investigation, visualization, and software; X.Z. to visualization and methodology; S.G. to investigation and software; Q.T. to methodology and software; X.H. to data curation and investigation; L.C. to methodology, validation, and investigation; and B.S. to project administration, resources, supervision, conceptualization, and funding acquisition.

## Supporting information



Supporting Information

Supplemental Video 1

## Data Availability

The data that support the findings of this study are available from the corresponding author upon reasonable request.
