# Supplementary material for: Additive Manufactured Magnetoelectric Wireless Retainer as the Periodontal Remodeler for Post‐Orthodontic Tissue Reconstruction and Relapse‐Inhibition
Source: Adv Sci (Weinh). 2025 Jul 23;12(39):e05020. doi: 10.1002/advs.202505020 (PMC12533409; doi:10.1002/advs.202505020)
Supplement: Supplementary file 1 — Supporting Information [file ADVS-12-e05020-s002.docx]

**Supplementary Figures**


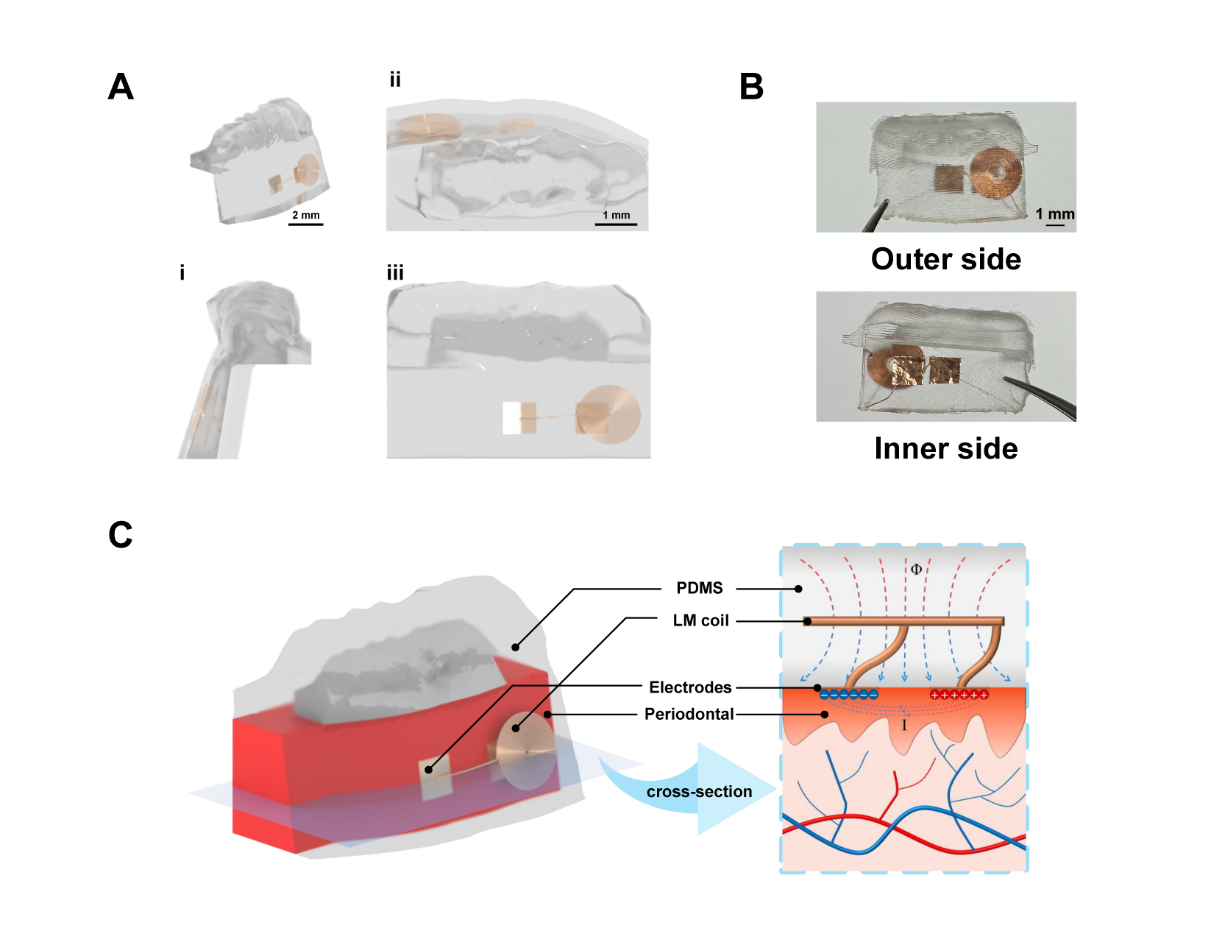


**Figure S1.** (A) Schematic diagram of FMOR structure. (i) - (iii) Ortho-isometric, top, side, (B) An optical image of outer side and inner side of FMOR for rats. (C) Schematic diagram of FMOR worn on periodontal.


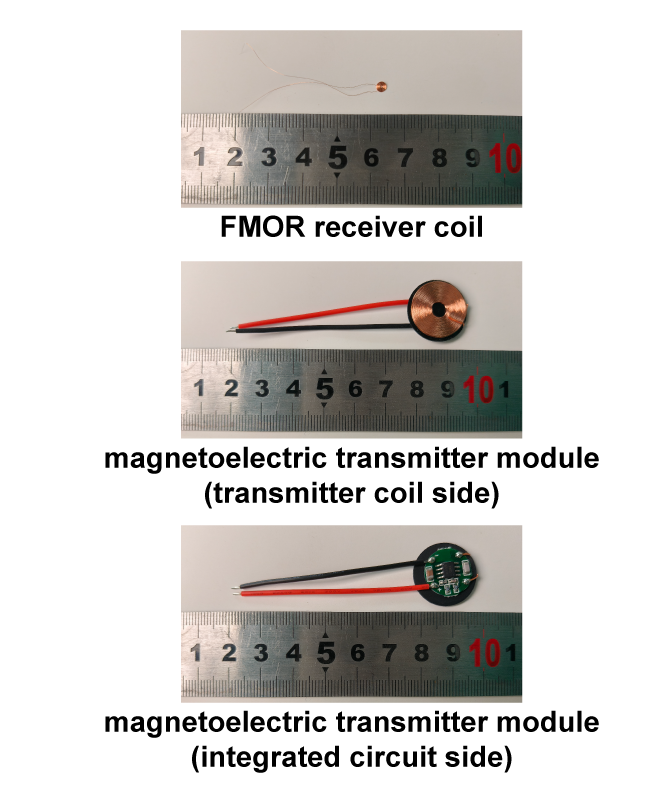


**Figure S2.** Photographs of components of FMOR and magnetoelectric transmitter.


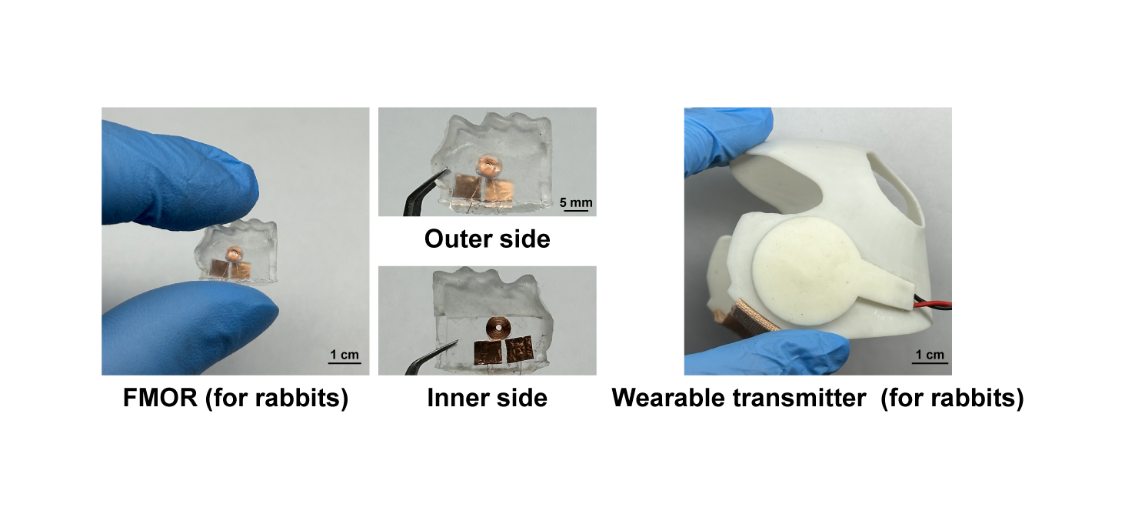


**Figure S3.** An optical image of FMOR and the wearable magnetoelectric transmitter for rabbits.


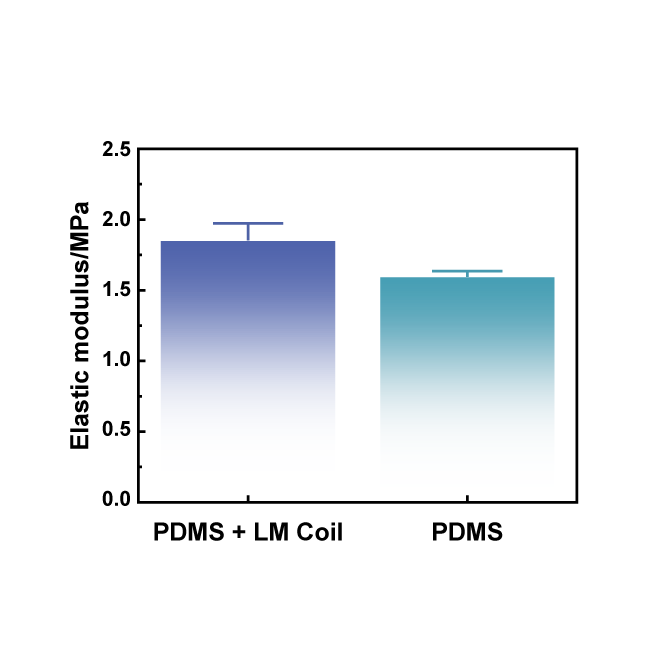


**Figure S4.** Mechanical properties of the FMOR.


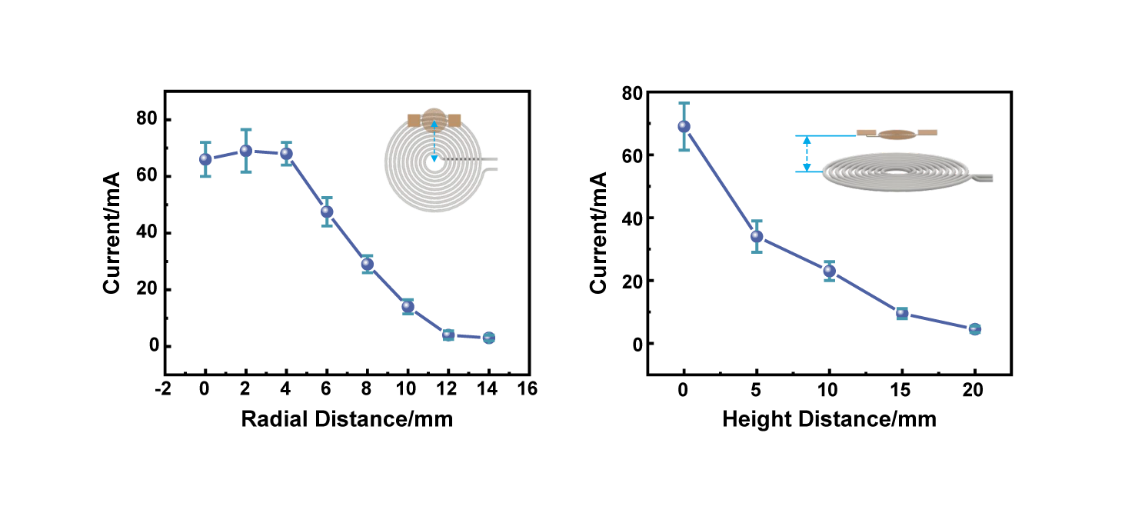


**Figure S5.** Current output of different radial distance (mm) and height distance (mm) between the FMOR receiving coil and the transmitting coil.


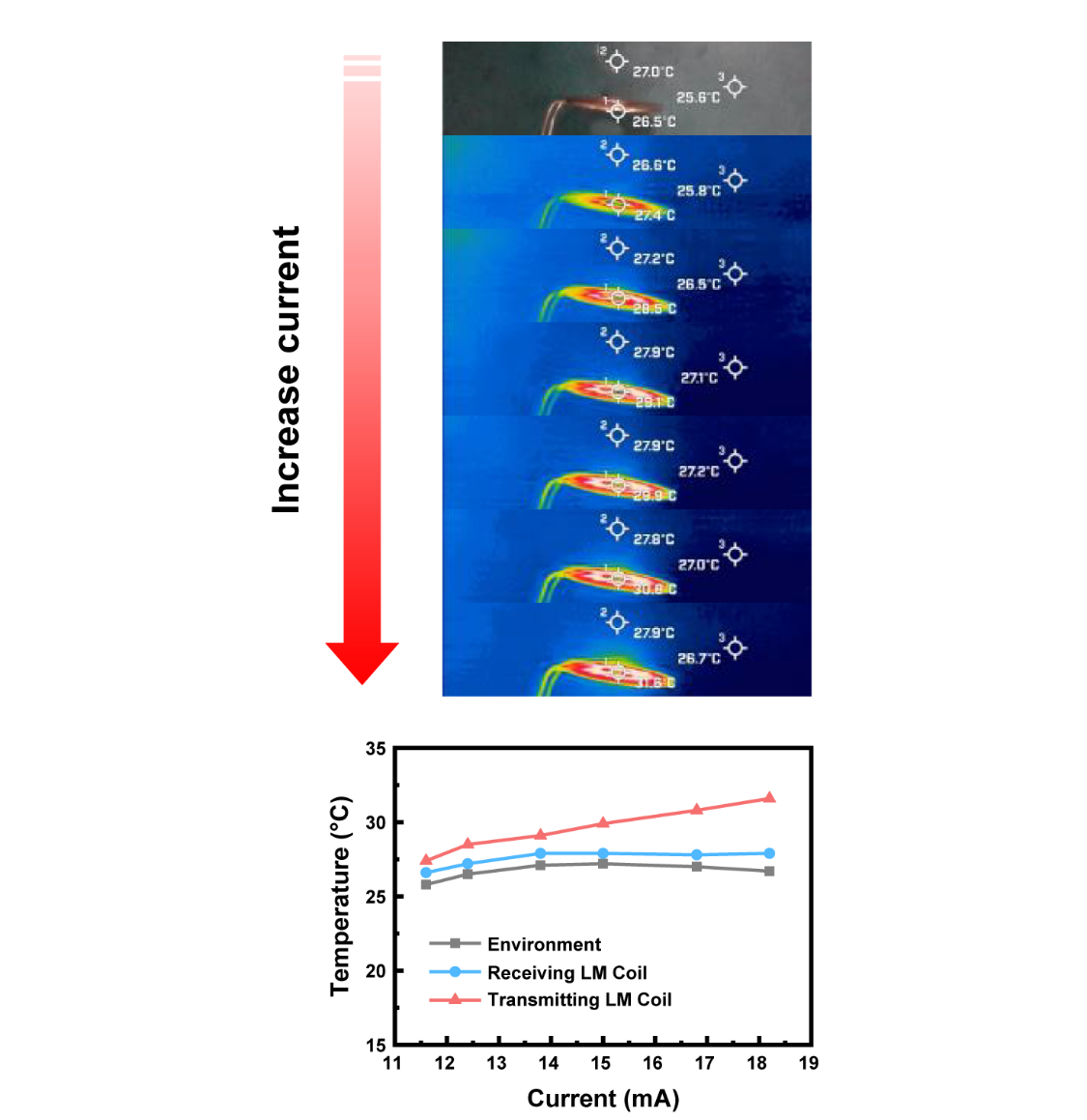


**Figure S6.** Real-time temperature detection with infrared images of FMOR receiving and transmitting LM coils at current output.


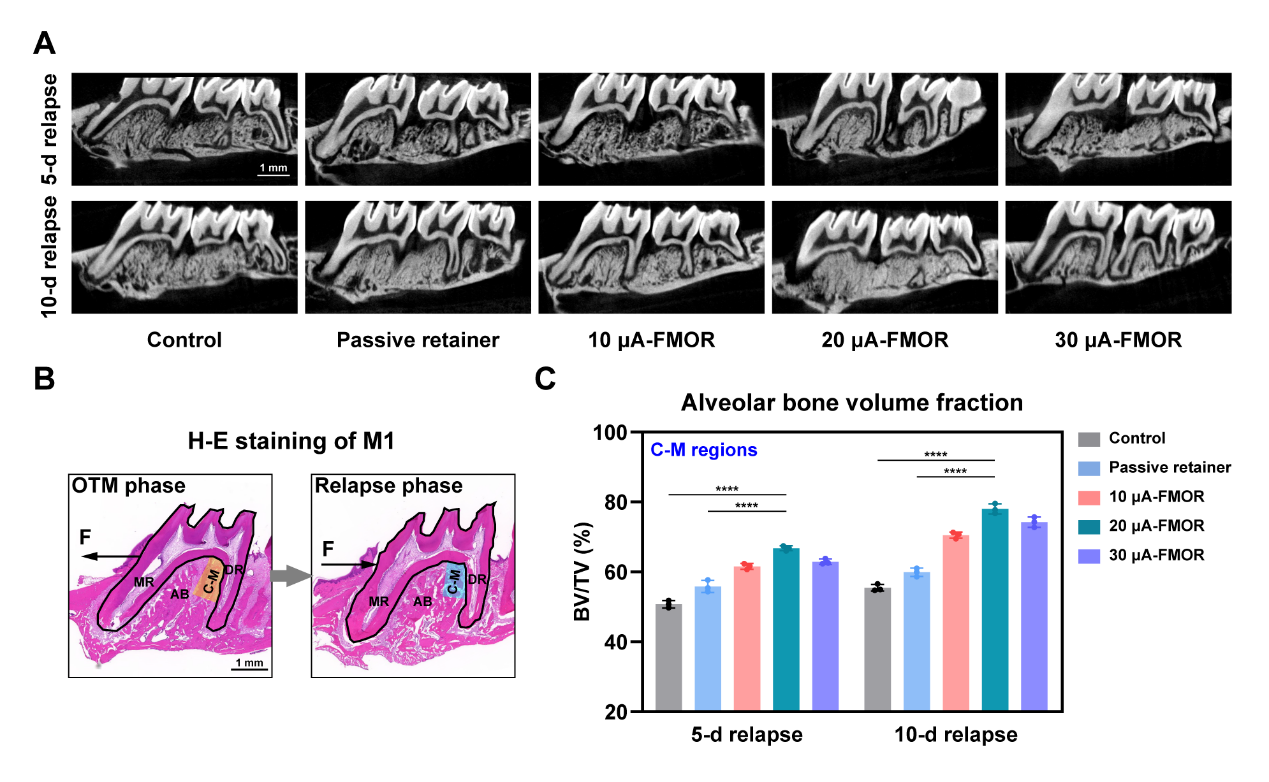


**Figure S7.** (A) Representative sagittal CT section images of rats’ dentition after 5 and 10 days of orthodontic relapse. Scale bar = 1 mm. (B) Schematic diagram of H&E staining showing the cervical mesial region of the distal root during orthodontic and relapse processes. Labels: M1 the first molar, MR mesial root, DR distal root, AB alveolar bone, C-M the cervical mesial region. The blue area indicates tension sides, the orange area indicates pressure sides. (C) Quantitative analysis of percent bone volume (BV/TV) of alveolar bone in the C-M regions. *p < 0.05, **p < 0.01, ***p < 0.001 and ****p <0.0001. Sample size (n = 3).


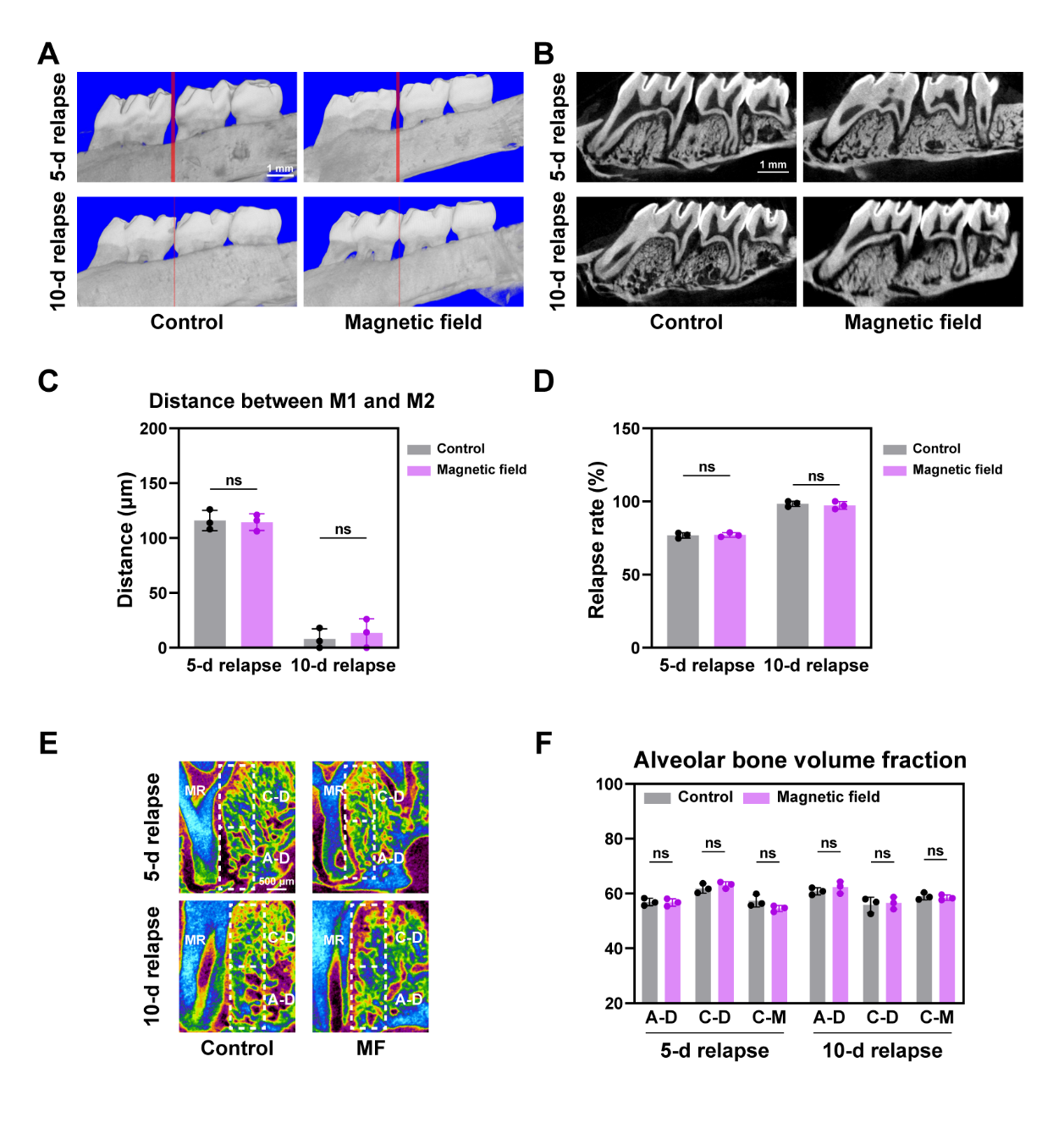


**Figure S8.** (A) Representative micro-CT images of rats’ dentition after 5 and 10 days of orthodontic relapse. The width of the red area is the distance between the first and second molars. Scale bar = 1 mm. (B) Representative sagittal CT section images of rats’ dentition after 5 and 10 days of orthodontic relapse. Scale bar = 1 mm. (C) and (D) Quantitative analysis of the distance between first and second molars and the relapse rate. Relapse rate = relapse distance / relapse distance at 0 d. (E) and (F) Micro–computed tomography images and quantitative analysis of percent bone volume (BV/TV) of alveolar bone in the A-D/C-D regions of MR and C-M regions of DR. *p < 0.05, **p < 0.01, ***p < 0.001 and ****p <0.0001. Sample size (n = 3).


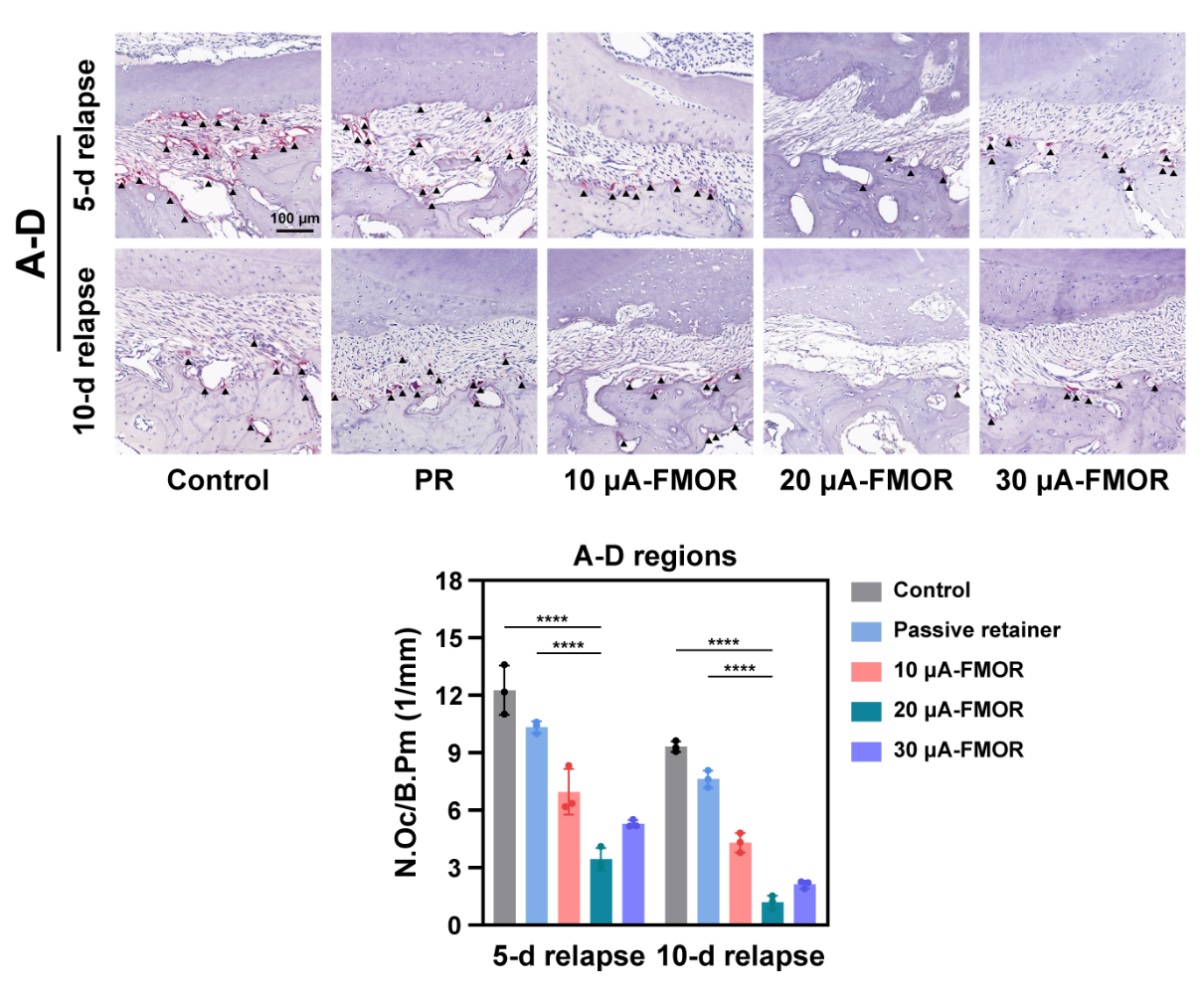


**Figure S9.** TRAP staining and quantitative analysis of the number of osteoclasts on histological sections of alveolar bone reconstruction in A-D region. Black triangles denote the TRAP-staining positive cells.


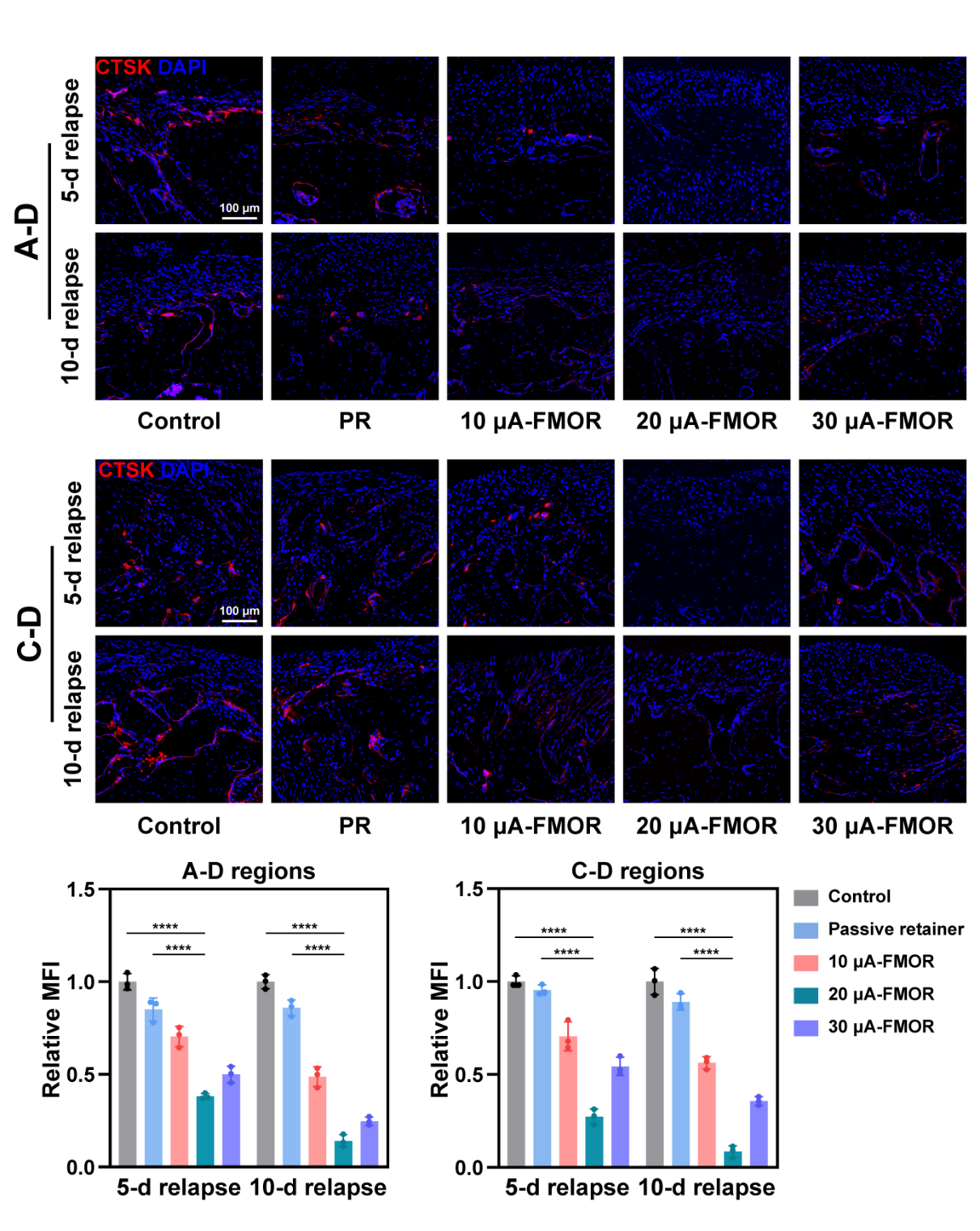


**Figure S10.** Representative immunofluorescence images and quantitative analysis of CTSK in histological sections of alveolar bone reconstruction in A-D and C-D regions.


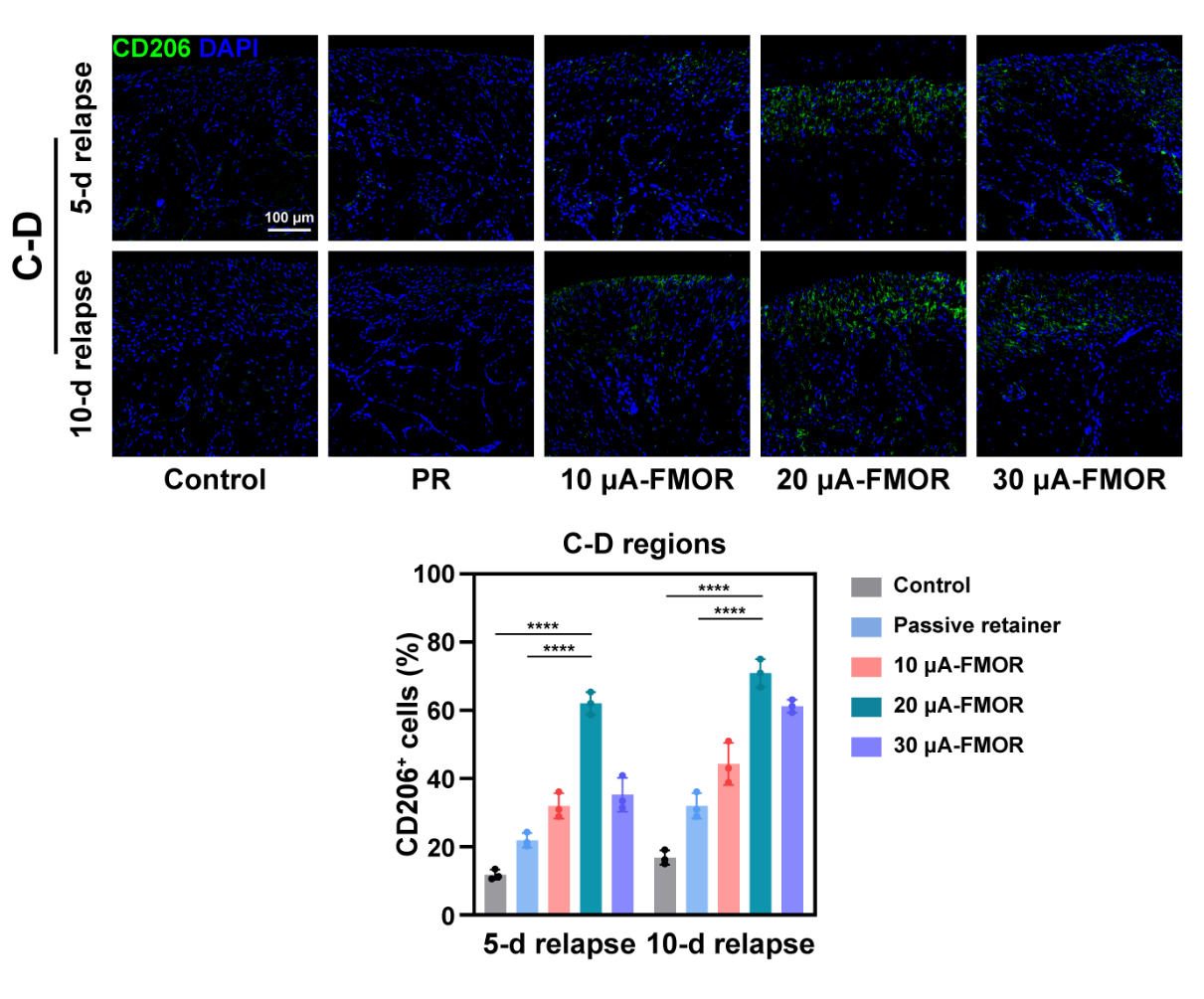


**Figure S11.** Representative immunofluorescence images and quantitative analysis of CD206 in histological sections of the pressure side of alveolar in C-D region.


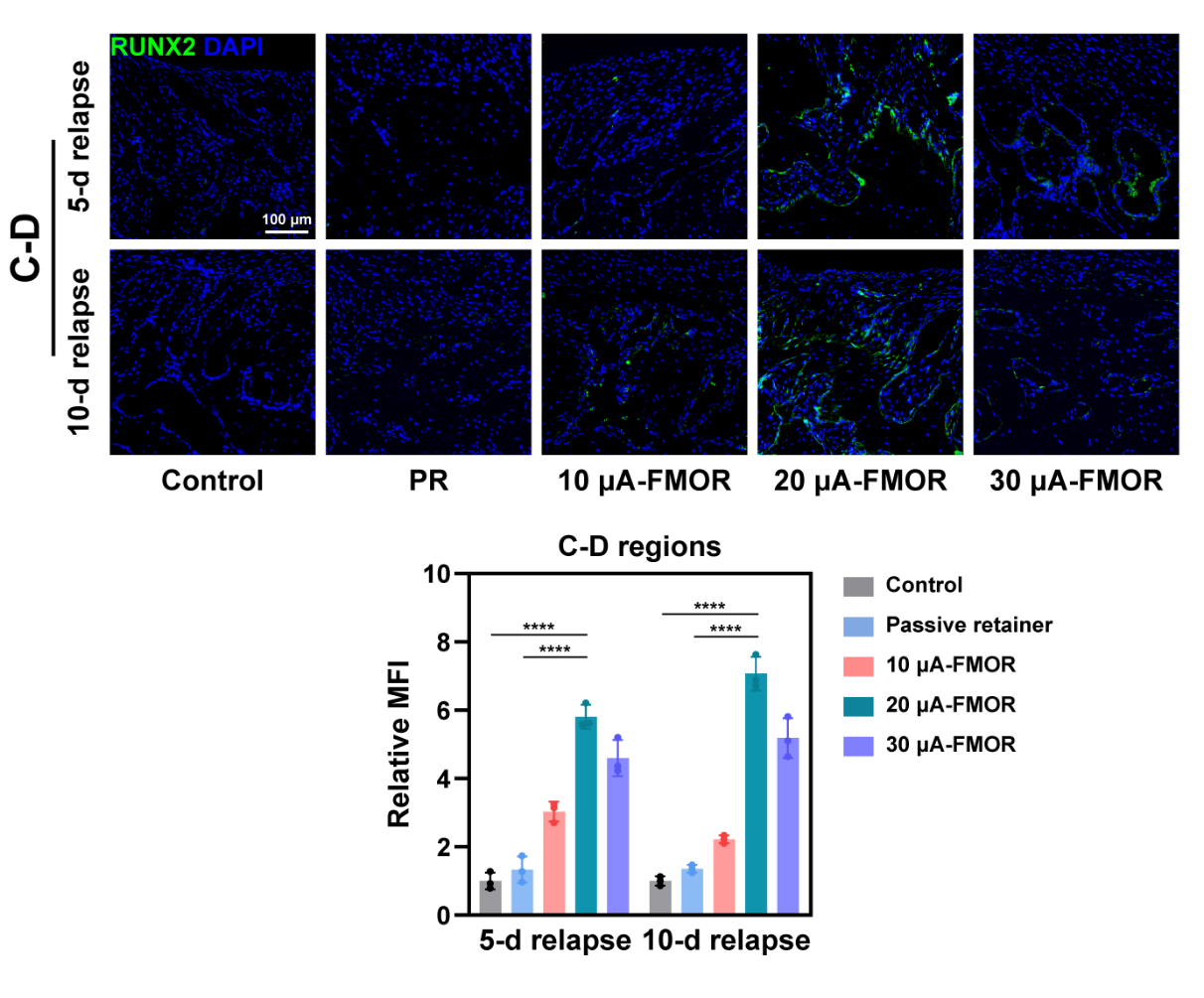


**Figure S12.** Representative immunofluorescence images and quantitative analysis of RUNX2 in histological sections of the pressure side of alveolar in C-D region.


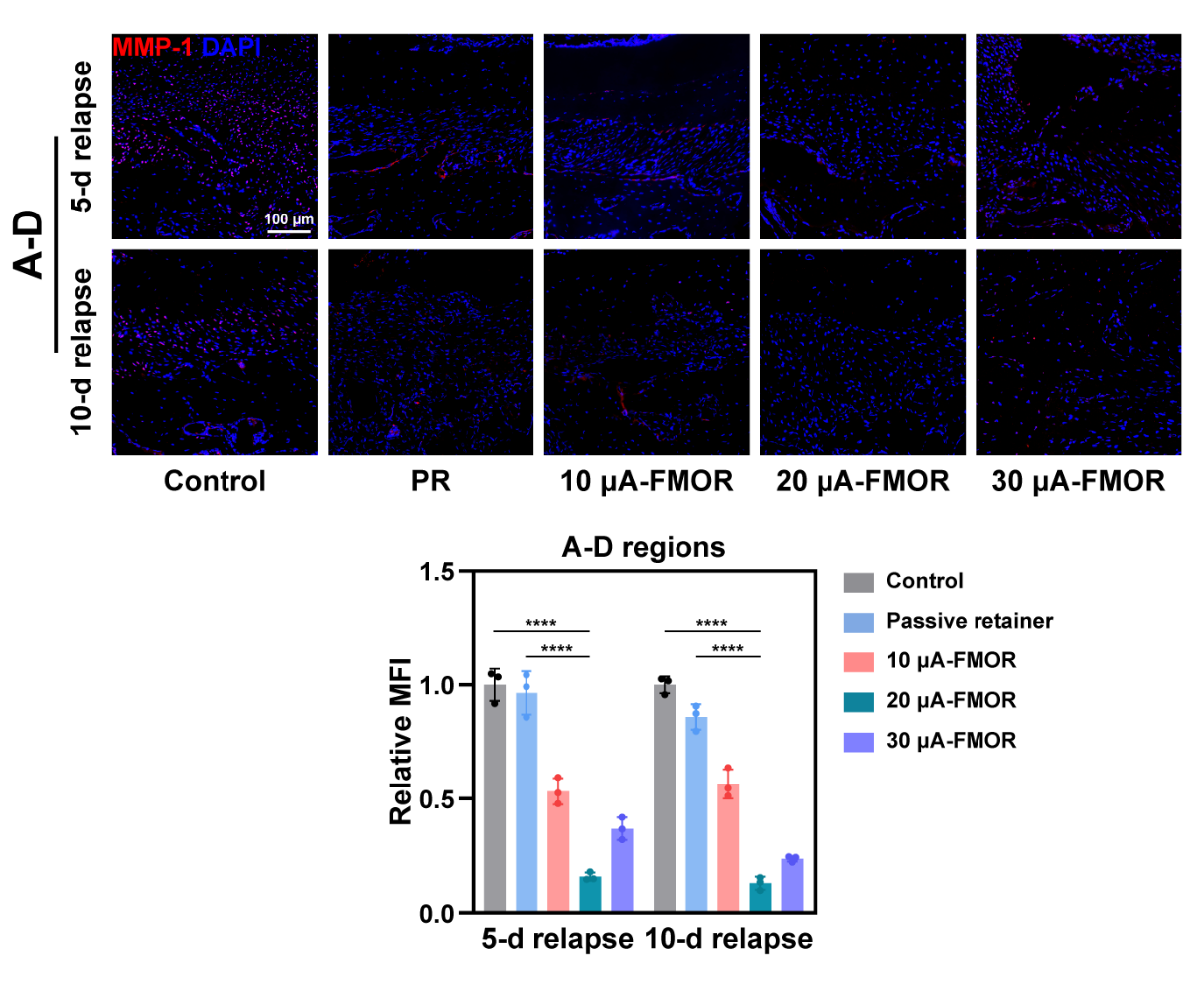


**Figure S13.** Representative immunofluorescence images and quantitative analysis of MMP-1 in histological sections of periodontal membrane reconstruction in A-D region.


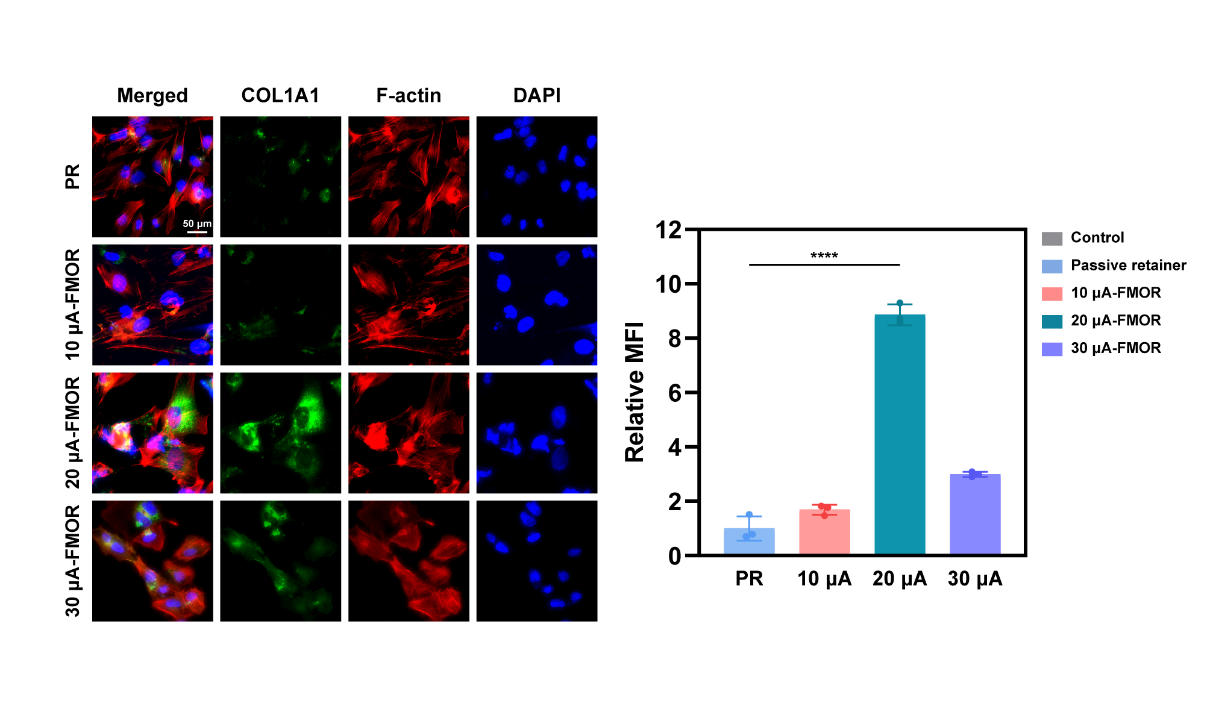


**Figure S14.** Representative immunostaining images and quantitative analysis of COL1A1 (green), F-actin (red) and cell nuclei (DAPI, blue) in h-FOBs co-cultured with PDLCs under wireless magnetoelectric output for 10 days.


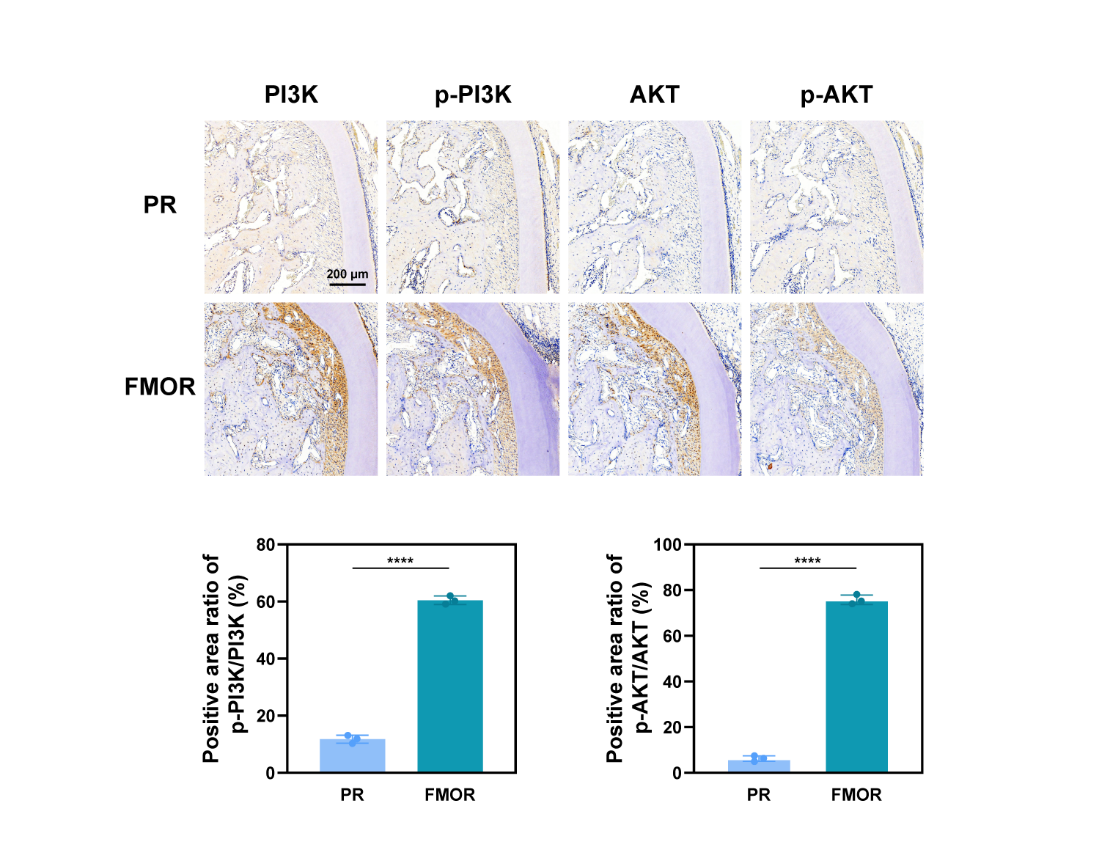


**Figure S15.** Representative immunohistochemical images and quantitative analysis of p-PI3K, PI3K, p-Akt, and Akt in histological sections of periodontal membrane. Scale bar = 200 μm. *p < 0.05, **p < 0.01, ***p < 0.001 and ****p <0.0001. Sample size (n = 3).

**Supplementary Tables**

**Table S1.** Materials used in production of magnetoelectric orthodontic retention system

| **Materials** | **Vendor or Source** |
| --- | --- |
| Polydimethylsiloxane (PDMS) | Huizhou Hongyejie Technology Co. |
| LM Coil | Xi'an Xinsai Jingtuo Technology Co. |
| Wireless transmitter | Huangshi Hanrui Trade Co. |
| UV curable resin | Formlabs |

**Table S2.** Young's Modulus of Commonly Used Transparent Solid Materials for Medical Use

|  | **Material** | **Young's modulus range** | **Literature source** |
| --- | --- | --- | --- |
| flexographic | PU | 0.9 MPa - 25 GPa | ^1^ |
|  | Ecoflex | 0.06 - 0.1 MPa | ^2^ |
|  | TPE | 0.1 - 100 MPa | ^3^ |
|  | PVA hydrogel | 10 - 100 kPa | ^4^ |
|  | PDMS | 0.5 - 2 MPa | ^5^ |
|  | This work | 1.6 - 1.8 MPa | / |
|  | TPU | 99 MPa | ^6^ |
| rigid | PETG | 2.2 GPa | ^7^ |
|  | Light-curing resins | 4.5 GPa | ^8^ |

**Table S3**. Primer sequences for qRT-PCR

| **Primer** | **Forward (5'-3')** | **Reverse (5'-3')** |
| --- | --- | --- |
| *CD163* | TTTGTCAACTTGAGTCCCTTCAC | TCCCGCTACACTTGTTTTCAC |
| *IL12* | ACCCTGACCATCCAAGTCAAA | TTGGCCTCGCATCTTAGAAAG |
| *VEGFα* | AGGGCAGAATCATCACGAAGT | AGGGTCTCGATTGGATGGCA |
| *TGFβ* | CTAATGGTGGAAACCCACAACG | TATCGCCAGGAATTGTTGCTG |
| *RUNX2* | TGGTTACTGTCATGGCGGGTA | TCTCAGATCGTTGAACCTTGCTA |
| *OPN* | CTGGTGCTCGTCCTCTACTAC | GGACACGAAGGTAAAGGTGAC |
| *COL1A1* | GAGGGCCAAGACGAAGACATC | CAGATCACGTCATCGCACAAC |
| *OCN* | CACACTCCTCGCCCTATTGG | TCAGCCAACTCGTCACAGTC |
| *MMP-1* | AAAATTACACGCCAGATTTGCC | GGTGTGACATTACTCCAGAGTTG |
| *MMP-2* | TACAGGATCATTGGCTACACACC | GGTCACATCGCTCCAGACT |
| *POSTN* | CTCATAGTCGTATCAGGGGTCG | ACACAGTCGTTTTCTGTCCAC |
| *ACTA2* | AAAAGACAGCTACGTGGGTGA | GCCATGTTCTATCGGGTACTTC |

**Table S4.** Information of antibodies

| **Primary Antibody** | **Company** | **Cat No.** |
| --- | --- | --- |
| CD206 Rabbit pAb | ABclonal, China | A8301 |
| CD86 Mouse mAb | Proteintech, China | 65165-1-Ig |
| RUNX2 Rabbit mAb | ABclonal, China | A11753 |
| Collagen I/COL1A1 Rabbit pAb | ABclonal, China | A1352 |
| Osteopontin/OPN Rabbit pAb | ABclonal, China | A1499 |
| Osteocalcin/OCN Rabbit mAb | ABclonal, China | A20800 |
| Cathepsin K/CTSK pAb | Proteintech, China | 11239-1-AP |
| MMP-1 Rabbit mAb | ABclonal, China | A22080 |
| Periostin/POSTN Rabbit mAb | ABclonal, China | A9009 |
| Anti-PI 3 Kinase p85 alpha mAb | Abcam | ab191606 |
| Phospho-PI3K p85 alpha mAb | Thermo | PA5104853 |
| Pan-Akt Rabbit pAb | ABclonal, China | A18120 |
| Phospho-Akt-S473 Rabbit mAb | ABclonal, China | AP1453 |
| CoraLite488-conjugated Goat Anti-Rabbit IgG(H+L) | Proteintech, China | SA00013-2 |
| CoraLite594 – conjugated Goat Anti-Rabbit IgG(H+L) | Proteintech, China | SA00013-4 |
| Cy3–conjugated Goat Anti-Mouse IgG(H+L) | Proteintech, China | SA00009-1 |

**Table S5.** Test kits

| **Name of the kit** | **Company** | **Cat No.** |
| --- | --- | --- |
| BCIP/NBT Alkaline Phosphatase Color Development Kit | Beyotime | C3206 |
| Alizarin Red S Staining Kit for Osteogenesis | Beyotime | C0148S |
| Tartrate-Resistant Acid Phosphatase (TRAP) Stain Kit | Solarbio | G1492 |
| HiScript III RT SuperMix for qPCR | Vazyme | R323-01 |
| ChamQ Universal SYBR qPCR Master Mix | Vazyme | Q711 |
| Hematoxylin and Eosin Staining Kit | Beyotime | C0105S |

**References**

1. Kampangsat, S., Kajornprai, T., Tangjatuporn, W., Suppakarn, N. & Trongsatitkul, T. Enhancing Tensile Modulus of Polyurethane-Based Shape Memory Polymers for Wound Closure Applications through the Addition of Palm Oil. *Polymers* **16**, 1941 (2024).

2. Zhu, G. *et al.* Fully Recyclable, Healable, Soft, and Stretchable Dynamic Polymers for Magnetic Soft Robots. *Advanced Functional Materials* **33**, 2300888 (2023).

3. Xiao, Y., Wang, W.-J., Li, B.-G. & Liu, P. High-Performance Olefin Thermoplastic Elastomer Based on Dynamically Cross-Linking Crystalline Macromers and Elastic Backbones. *Macromolecules* **57**, 1788–1794 (2024).

4. Gu, L. *et al.* AIE-active PVA/berberine antibacterial hydrogel for wound healing, visual monitoring pH and dehydration. *Biomaterials* **323**, 123432 (2025).

5. Liu, Y. *et al.* Contribution of m5C RNA Modification-Related Genes to Prognosis and Immunotherapy Prediction in Patients with Ovarian Cancer. *Mediators of Inflammation* **2023**, 1400267 (2023).

6. Ho, C.-T., Huang, Y.-T., Chao, C.-W., Huang, T.-H. & Kao, C.-T. Effects of different aligner materials and attachments on orthodontic behavior. *Journal of Dental Sciences* **16**, 1001–1009 (2021).

7. Chen, P.-Y., Huang, H.-L., Yu, J.-H. & Hsu, J.-T. Effects of Attachment Design and Aligner Material on Mandibular Canine Distal Bodily Movement in Aligner Treatment. *J. Med. Biol. Eng.* **44**, 777–787 (2024).

8. Zimmermann, M., Ender, A., Egli, G., Özcan, M. & Mehl, A. Fracture load of CAD/CAM-fabricated and 3D-printed composite crowns as a function of material thickness. *Clin Oral Invest* **23**, 2777–2784 (2019).
